# Racial, ethnic, and gender differences in obesity and body fat distribution: An All of Us Research Program demonstration project

**DOI:** 10.1371/journal.pone.0255583

**Published:** 2021-08-06

**Authors:** Jason H. Karnes, Amit Arora, Jianglin Feng, Heidi E. Steiner, Lina Sulieman, Eric Boerwinkle, Cheryl Clark, Mine Cicek, Elizabeth Cohn, Kelly Gebo, Roxana Loperena-Cortes, Lucila Ohno-Machado, Kelsey Mayo, Steve Mockrin, Andrea Ramirez, Sheri Schully, Yann C. Klimentidis

**Affiliations:** 1 Department of Pharmacy Practice and Science, College of Pharmacy, University of Arizona, Tucson, Arizona, United States of America; 2 Department of Epidemiology and Biostatistics, Mel and Enid Zuckerman College of Public Health, University of Arizona, Tucson, Arizona, United States of America; 3 Vanderbilt University Medical Center, Nashville, Tennessee, United States of America; 4 University of Texas Health Science Center at Houston, Houston, Texas, United States of America; 5 Brigham and Women’s Hospital, Boston, Massachusetts, United States of America; 6 Mayo Clinic, Rochester, Minnesota, United States of America; 7 Hunter College, City University of New York, New York, New York, United States of America; 8 National Institutes of Health, Bethesda, Massachusetts, United States of America; 9 University of California San Diego Health, San Diego, California, United States of America; 10 BIO5 Institute, University of Arizona, Tucson, Arizona, United States of America; Emory University, UNITED STATES

## Abstract

Differences in obesity and body fat distribution across gender and race/ethnicity have been extensively described. We sought to replicate these differences and evaluate newly emerging data from the All of Us Research Program (AoU). We compared body mass index (BMI), waist circumference, and waist-to-hip ratio from the baseline physical examination, and alanine aminotransferase (ALT) from the electronic health record in up to 88,195 Non-Hispanic White (NHW), 40,770 Non-Hispanic Black (NHB), 35,640 Hispanic, and 5,648 Asian participants. We compared AoU sociodemographic variable distribution to National Health and Nutrition Examination Survey (NHANES) data and applied the pseudo-weighting method for adjusting selection biases of AoU recruitment. Our findings replicate previous observations with respect to gender differences in BMI. In particular, we replicate the large gender disparity in obesity rates among NHB participants, in which obesity and mean BMI are much higher in NHB women than NHB men (33.34 kg/m^2^ versus 28.40 kg/m^2^ respectively; p<2.22x10^-308^). The overall age-adjusted obesity prevalence in AoU participants is similar overall but lower than the prevalence found in NHANES for NHW participants. ALT was higher in men than women, and lower among NHB participants compared to other racial/ethnic groups, consistent with previous findings. Our data suggest consistency of AoU with national averages related to obesity and suggest this resource is likely to be a major source of scientific inquiry and discovery in diverse populations.

## Introduction

Type-2 diabetes and cardiovascular disease are among the most pressing health issues of our time, responsible for major mortality and financial burdens across the globe [1–4]. Although these affect nearly all human populations, these diseases and their risk factors, such as obesity and fat distribution, exhibit notable disparities across different racial/ethnic groups in the United States (US) and globally [5, 6]. For example, data from the National Health and Nutrition Examination Survey (NHANES) shows that the prevalence of obesity is highest among Non-Hispanic Black women, and that a large gender disparity in obesity exists among Non-Hispanic Black individuals compared with other groups [5]. The prevalence of non-alcoholic fatty liver disease (NAFLD) also exhibits disparities across racial/ethnic groups, generally being highest among Hispanic individuals, and lowest among non-Hispanic Black individuals [7].

The All of Us research program (AoU) is a national-scale effort to collect health-related information in one million US residents [8]. Here, we used the first interim release of the data to replicate previous findings in order to assess the suitability, breadth, depth, and quality of AoU data. Our primary objective was to replicate previous findings on racial, ethnic, and gender disparities in obesity and body fat distribution, including levels of alanine aminotransferase (ALT), a surrogate measure of hepatic steatosis. Our secondary objective was to examine how nationally representative AoU participants are with regard to obesity and body fat distribution, given the potential biases introduced by the recruiting methodology and other factors. This information could help inform future epidemiological studies using the AoU data with respect to assessing potential biases and the generalizability of findings.

## Methods

### All of Us Research Program (AoU) design and data collection

AoU is a large, collaborative initiative sponsored by the National Institutes of Health (NIH) to recruit one million or more individuals willing and able to answer questionnaires (Participant-provided information [PPI]), provide biospecimens and physical measurements, share their electronic health record (EHR) data, and authorize re-contact. AoU is designed as a longitudinal cohort study open to all individuals (age>18) currently residing in the US or US territory. A network of 22 healthcare provider organizations led recruitment efforts, with a particular focus on recruitment of individuals in groups that are typically underrepresented in biomedical research [8–10]. The program also recruited direct volunteers open to any eligible individuals (S1 File). While no recruitment quotas are used, enrichment of groups that are typically underrepresented in biomedical research is accomplished through an number of recruitment outreach approaches, including targeted advertisement, personal interest groups, and community outreach at healthcare provider organizations or direct volunteers partner sites. Participants that were included in this manuscript were recruited between 2018 and 2020. Only individuals without the decisional capacity to consent, prisoners at time of enrollment, and individuals under the age of 18 were excluded from eligibility in AoU at the time of this analysis. All participants provide electronic informed consent. The All of Us Research Program Institutional Review Board has established that Registered Tier data available on the Researcher Workbench (https://workbench.researchallofus.org/) meets criteria for non-Human Subjects Research and this demonstration project did not require IRB review.

The AoU Registered Tier data available on the Research Hub contains data from participants who have consented to be involved in the All of Us Research Program, including data from electronic health records, surveys, and physical measurements. All data available to researchers has had direct identifiers removed and has been further modified to minimize re-identification risks. This includes removing all explicit identifiers in both EHR and PPI, all free-text fields, geolocation data smaller than U.S. state level, living situations, race and ethnicity subcategories, active duty military status, cause of death, and diagnosis codes subject to public knowledge. Additionally, the following demographic fields are generalized: race and ethnicity, education, employment, and information regarding sex at birth, gender identity, and sexual orientation. Also, all dates are systematically shifted backwards by a random number between 1 and 365, and data from participants over the age of 89 are removed. AoU data will be accessed for research strictly using the Researcher Workbench. (researchallofus.org). External data can be brought into this secure environment; however, researchers are restricted from importing any individually identifiable information and from row-level linkage of the external data. Data searches, cohort building, and analysis will solely take place on the Researcher Workbench, a secure cloud-based resource with statistical analysis software available for use with AoU data. Researchers are granted access to the Researcher Workbench after creating an account, including setting up two-factor authentication, validating their eRA Commons ID, completing the AOU Responsible Conduct of Research training, and signing a Data Use and Registration Agreement that prohibits any re-identification of AoU participants.

Participants self-report their racial and ethnic identity by responding to the question: Which categories describe you? (Select all that apply): 1) American Indian and Alaska Native; 2) Black, African American, or African; 3) Asian; 4) Hispanic, Latino, or Spanish; 5) Middle Eastern or North African; 6) Native Hawaiian or Other Pacific Islander; 7) White; 8) None of these describe me; 9) Prefer Not To Answer (S2 and S3 Files). Due to privacy methodology, PPI-derived racial/ethnic categories were condensed to White; Black; Asian; Hispanic, Latino or Spanish; Other (if one of options 5–6 or None); Two or more races (if multiple options 1–7 selected); and Prefer not to answer. Data for American Indian and Alaska Native race/ethnicity were not accessible in registered tier AllofUs data due to privacy methodology outlined above. Only participants included in the White, Black, Asian, and Hispanic participant race/ancestry categories were included in the present analysis.

Self-identified gender was assessed at the baseline visit using PPI-derived gender based on the question “What terms best express how you describe your gender identity? (Check all that apply): 1) Man; 2) Woman; 3) Non-binary; 4) Transgender; 5) None of these describe me, and I’d like to consider additional options; 6) Prefer not to answer. Due to privacy methodology, We only considered those reporting as men and women in our analyses since privacy methodology outlined above precluded availability of all other gender identities. A standardized set of physical measurements were obtained from participants at the baseline visit (S1 File). Height, weight, waist circumference (WC), and hip circumference measurements were obtained according to AoU baseline visit protocols. Levels of alanine aminotransferase (ALT) were obtained from the EHR records of participants. ALT measures were taken from the EHR starting in 2015, with all prior ALT measures being excluded. Heavy drinkers (≥14 drinks per week) were determined based on PPI survey questions about consumption of alcoholic drinks (S4 and S5 Files), and were excluded from analyses of ALT since elevated ALT could be attributable to high alcohol consumption [11, 12]. Additional details regarding AoU design, anthropomorphic measurements, and EHR laboratory results are available (S1 File).

### Statistical analyses

**Fig 1:**
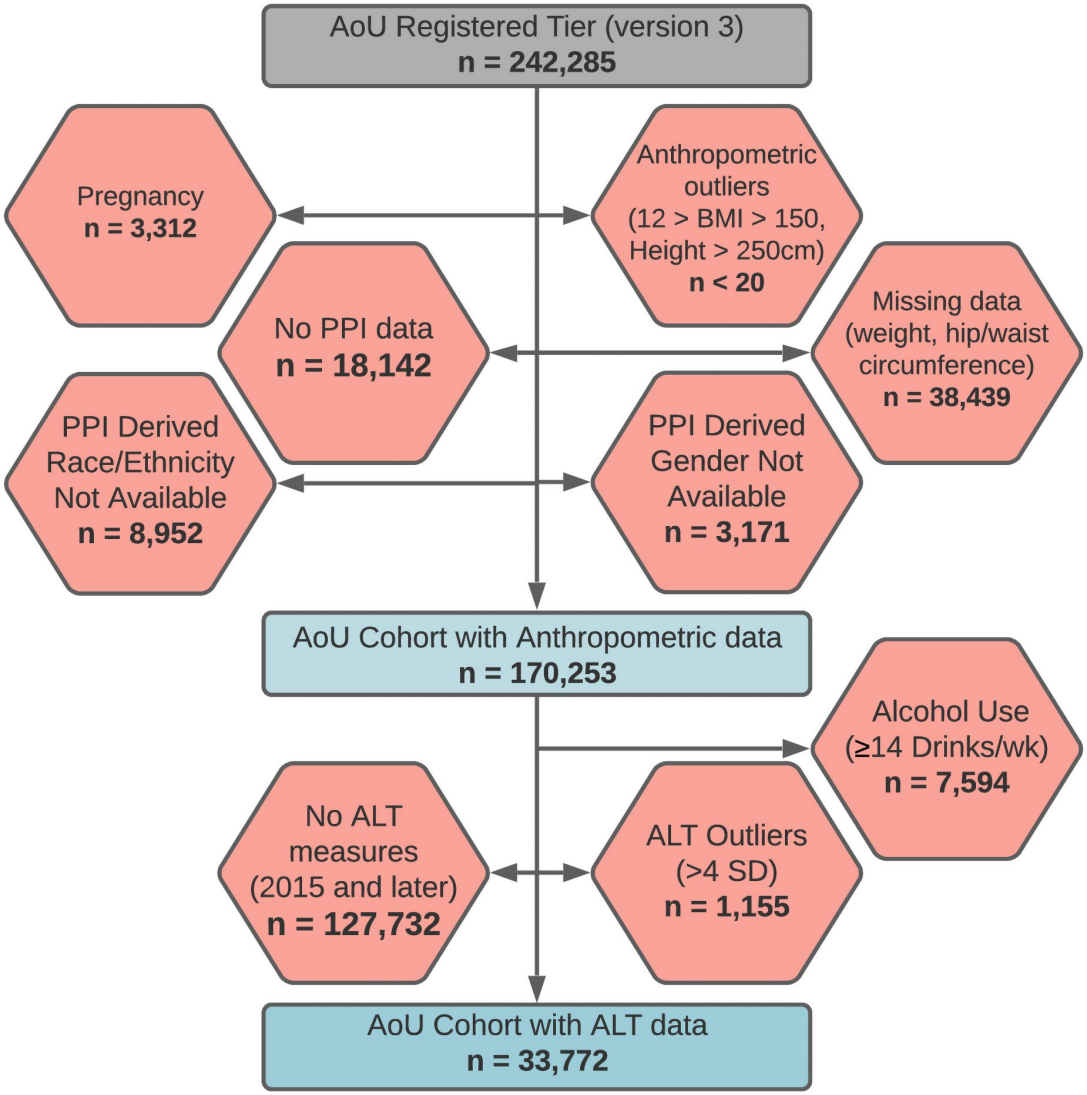
Creation of AoU cohort with anthropometric data and AoU cohort with ALT data from all participants in AoU registered tier data. Participants were removed from AoU Registered Tier data for the current study based on exclusion criteria, outlying observations, availability of data, and responses to PPI-based questions regarding race/ethnicity and gender. All participants in the AoU cohort with ALT data are included in the AoU cohort with anthropometric data. ALT indicates Alanine Aminotransferase; AoU, All of Us Research Program; BMI, body mass index; cm, centimeters; PPI, participant-provided information; SD, standard deviation; wk, week.

AoU data is constituted by a patchwork of datasets, including PPI, physical measurement, and EHR datasets with varying numbers of total patients [8]. All participant data from the Registered Tier dataset were used if the requisite data for statistical analysis existed on a participant, ensuring the maximum number of individuals in each analysis described below (Fig 1). The distribution of anthropomorphic measurements were examined visually and major outliers for height >250 cm (n≤20) were completely removed. Similarly, the distribution of calculated anthropomorphic indices including body mass index (BMI) and waist-to-hip ratio (WHR) were visually examined and major outliers were removed: BMI>150 kg/m^2^ (n≤20), BMI<12 kg/m^2^ (n≤20), WHR<0.2 (n≤20), and WHR>6 (n≤20). In order to avoid biases in anthropomorphic measurements, patients who self-reported as pregnant were excluded from all analyses. Obesity was defined as having a BMI≥30kg/m^2^. Outliers for ALT data were defined as those individuals with a value greater than 4 standard deviations above the mean, as previously defined [13], after removing extreme, biologically implausible values (ALT>150,000 IU/L). Only ALT measurements acquired in 2015 and afterwards we used in this analysis and the most recent ALT measurement was used if multiple measurements were available on a participant. Participants were excluded from ALT analyses if multiple ALT measures were available at the same date and time but with a different ALT result. Due to privacy restrictions related to AoU publications policy, no single individual or groups of individuals below 20 are reported, and outlying observations are not plotted on boxplots.

We performed two analyses to assess the representativeness of the AoU population to the U.S. population and to account for potential biases due to the AoU recruitment approach. Firstly, we generated age-adjusted obesity prevalence estimates for all gender and race/ethnicity groups in AoU, NHANES 2015–2016, and NHANES 2017–2018. When evaluating obesity prevalence by gender/race/ethnicity group, we directly adjust for selection bias by matching the distribution of the selection variable of AoU to that of the 2010 US Census using six age groups (18–29, 30–39, 40–49, 50–59, 60–69, and 70 and over) [5, 14]. For each gender and race/ethnicity subset, we use proportions of the six age groups in the US population for each subset as reference weights to adjust the weights of AoU and NHANES samples, and then calculate the weighted means and variances of obesity. Kish’s effective sample sizes are used for the confidence interval calculation. We also performed t-tests for differences in obesity prevalence within gender /race/ethnicity groups between AoU, NHANES 2015–2016, and NHANES 2017–2018. Secondly, we employed a pseudo weight approach to produce age, gender, and race/ethnicity adjusted estimates for demographic and anthropometric variables as well as ALT [15]. The common variables age, gender, and race/ethnicity plus the quadratic term of age are used to obtain the pseudo weights by using logistic regression on the combined sample (NHANES 2015–2016 and 2017–2018 combined sample and the general AoU sample, or NHANES combined sample and the AoU with ALT sample), and these weights are then used to estimate the means of other variables (height, weight, BMI, WC, obesity and ALT). For pseudo weight analysis, AoU data was restricted to the same time period (2015–2018). AoU participation was restricted to individuals at least 18 years of age and an age cutoff of 18 was implemented in NHANES data for pseudo weighting.

We created boxplots to visually examine distributions of BMI, WC, WHR, and ALT by race/ethnicity and by gender. Student’s t-tests were used to test for differences in continuous variables, including BMI, WC, WHR, and ALT, between men and women within race/ethnicity categories. We conducted linear regressions to model variables contributing significantly to variability in BMI and ALT with race/ethnicity, gender, age, age^2^, and BMI (for ALT modelling) included as independent variables. Additional linear regressions were performed with gender x race interaction terms. Significance was determined at an alpha level of 0.05 for both t-tests and linear regressions. A normal distribution was assumed based on the large numbers of participants included for each subgroup. This demonstration project underwent code review by AoU Data and Research Center personnel to ensure validity and reproducibility of results. All statistical analyses were performed in R version 3.6.2 within the AoU Researcher Workbench Jupyter Notebook. The code used for this demonstration project is available within the Researcher Workbench at https://workbench.researchallofus.org/workspaces/aou-rw-54ae5687/racialethnicdifferencesanthropolipidalt/notebooks/Notebook_addressingComments.ipynb and https://workbench.researchallofus.org/workspaces/aou-rw-54ae5687/racialethnicdifferencesanthropolipidalt/notebooks/AoU_DemoProject.ipynb

## Results

A total of 242,285 participants were included in the AoU Registered Tier data at the time of code review. Of the total participants in the dataset, 224,143 had the necessary PPI-derived data on race/ethnicity and gender for our analysis. Of these participants, 170,253 were used in the analysis of anthropomorphic measurements and 33,772 were used in the analysis of ALT measurements. Our analyses of anthropomorphic measurements, BMI, and WHR included a total of 88,195 Non-Hispanic White (NHW), 40,770 Non-Hispanic Black (NHB), 35,640 Hispanic, and 5,648 Asian participants. The mean age of participants was higher among NHW and NHB participants than among Hispanic and Asian participants (Table 1).

**Table 1:** Characteristics of age, anthropometric traits and ALT levels by race/ethnicity and gender^a^.

Race/Ethnicity and Gender	N	Age (years)	BMI (kg/m^2^)	Prevalence of obesity^b^	Waist Circumference (cm)	Hip Circumference (cm)	WHR	N^c^	ALT (IU/L)
NHW Women	53,136	54.3 (16.8)	29.09 (7.69)	0.36	91.34 (17.9)	108.6 (16.2)	0.84 (0.09)	12,550	22.9 (19.44)
NHW Men	35,059	57.1 (16.8)	29.02 (6.14)	0.33	101.3 (16.2)	106.5 (12.4)	0.95 (0.09)	6,870	27.9 (21.8)
NHB Women	22,967	50.2 (14.6)	33.34 (9.11)	0.59	100.5 (18.6)	115.4 (18)	0.87 (0.1)	4,305	18.8 (15.55)
NHB Men	17,803	50.9 (13.7)	28.4 (7.13)	0.32	96.94 (17.7)	105.2 (14.1)	0.92 (0.11)	2,043	25.3 (20.98)
Hispanic Women	23,435	45.7 (15.6)	31.06 (7.43)	0.49	94.83 (16.8)	109.8 (15.3)	0.86(0.09)	5,108	25.8 (26.63)
Hispanic Men	12,205	46.0 (15.8)	29.75 (6.69)	0.40	100.1 (16.6)	105.4 (12.9)	0.95 (0.09)	2,118	31.1 (27.2)
Asian Women	3,405	43.0 (17)	24.49 (4.98)	0.13	79.3 (12.5)	96.34 (10.9)	0.82 (0.08)	495	21.1 (18.9)
Asian Men	2,243	44.8 (17.2)	26.12 (4.62)	0.17	89.7 (13.1)	99.43 (9.49)	0.90 (0.11)	283	30.5 (24.4)

ALT indicates alanine aminotransferase; BMI, body mass index; cm, centimeters; IU/L, international units per liter; NHB, Non-Hispanic Black; NHW, Non-Hispanic White; WHR, waist-to-hip ratio.

^a^Continuous variables are presented as mean (standard deviation).

^b^Age-adjusted obesity prevalence.

^c^Number of individuals with available ALT data that were included form the AoU EHR dataset.

BMI was highest among NHB women (mean 33.32 kg/m^2^) and lowest among Asian women (mean 24.49 kg/m^2^) (Table 1). The gender difference in BMI was most pronounced among NHB, whereby NHB women had higher BMI than NHB men (33.34 kg/m^2^ versus 28.40 kg/m^2^ respectively, p<2.22x10^-308^) (Table 2). This pattern was similar among Hispanics and almost absent in NHWs (Table 1 and S1 Table in S1 File). Multi-variable regressions indicated an increased BMI with increasing age (β = 0.33[standard error 0.01], p<2.22x10^-308^), in women versus men (β = 1.45[0.04], p<2.22x10^-308^), NHB versus NHW participants (β = 1.89[0.05], p<2.22x10^-308^) and Hispanic versus NHW participants (β = 1.45[0.05], p = 2.04x10^-198^). Significant race/ethnicity x gender interaction terms were also observed for all racial/ethnic groups (Table 2).

**Table 2:** Results of multivariable linear regression models for body mass index.

Covariate	β^a^	SE^a^	L95^a^	U95^a^	P Value^a^
**Multivariable Regression for BMI (R^2^ = 0.051)**					
Age (years)	0.33	0.01	0.32	0.34	<2.22x10^-308^
Age^2^ (years^2^)	-0.0032	0.0001	-0.0033	-0.0030	<2.22x10^-308^
Women	1.45	0.04	1.38	1.52	<2.22x10^-308^
NHB	1.89	0.05	1.80	1.98	<2.22x10^-308^
Hispanic	1.45	0.05	1.35	1.54	2.04x10^-198^
Asian	-3.71	0.10	-3.92	-3.51	2.06x10^-285^
Multivariable Regression for BMI with Race/Ethnicity*Gender Interaction Terms (R^2^ = 0.070)					
Age (years)	0.34	0.01	0.33	0.35	<2.22x10^-308^
Age^2^ (years)	-0.003	0.0001	-0.003	-0.003	<2.22x10^-308^
Women	0.01	0.05	-0.09	0.11	0.86
NHB	-1.01	0.07	-1.15	-0.88	4.60x10^-49^
Hispanic	0.66	0.08	0.50	0.81	7.61x10^-17^
Asian	-2.78	0.16	-3.10	-2.47	3.11x10^-67^
Women*NHB	5.02	0.09	4.84	5.19	<2.22x10^-308^
Women*Hispanic	1.29	0.10	1.10	1.48	1.58x10^-40^
Women*Asian	-1.57	0.21	-1.97	-1.17	2.62x10^-14^

BMI, body mass index; L95, lower limit of 95% confidence interval for beta; NHB, Non-Hispanic Black; NHW, Non-Hispanic White; SE, standard error; U95, upper limit of 95% confidence interval for beta.

^a^P values, betas, and standard errors, L95, and U95 for betas were generated using linear regressions with BMI as the outcome and age, age^2^, gender, and race/ethnicity as covariates.

In all regressions, men and NHW race were used as reference groups. Multiple linear regressions were performed with and without race/ethnicity*gender interaction terms. Significance was determined at an alpha level of 0.05. A normal distribution was assumed based on the large numbers of participants included for each subgroup. P values lower than 2.22x10^-308^ were not calculated.

**Fig 2:**
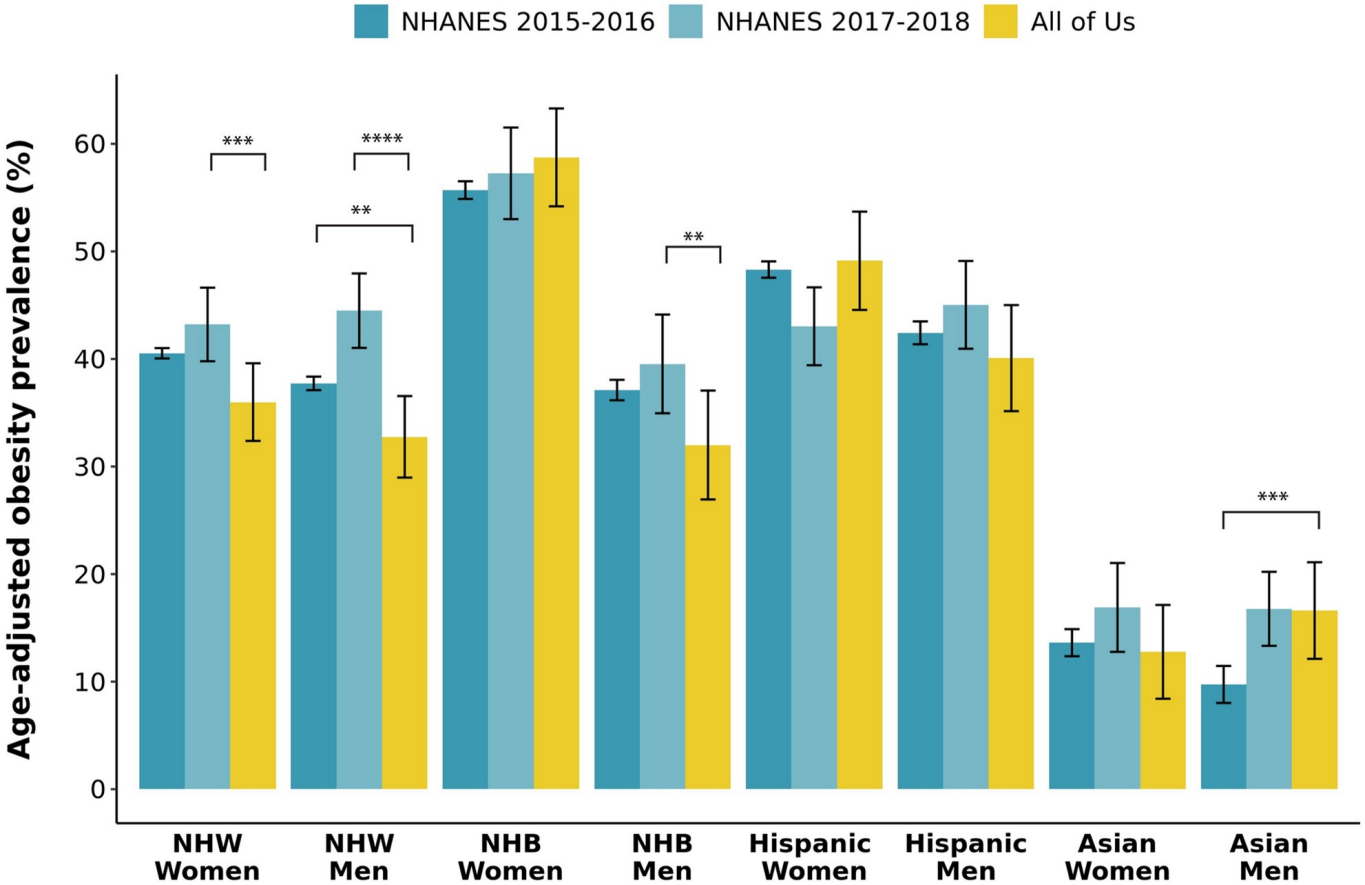
Age-adjusted prevalence of obesity by race/ethnicity and gender in all of us research program compared to NHANES 2015–2016 and 2017–2018 data. Race/ethnicity and gender groups are represented on the X axis and proportion of individuals with obesity adjusted for age is represented on the Y axis. For each gender and race/ethnicity subset, proportions of six age groups in the US population for each subset were used as reference weights to obtain the weights for AoU and NHANES cohorts, to calculate the weighted mean, and to calculate the variance where Kish’s effective sample size is used to calculate the confidence interval. T-tests were then performed to evaluate differences in obesity prevalence within gender/race/ethnicity groups between AoU, NHANES 2015–2016, and NHANES 2017–2018. Only p values less than 0.01 are indicated. ** p<0.01; *** p<0.001; **** p<0.0001. NHANES indicates National Health and Nutrition Examination Survey; NHB, Non-Hispanic Black; and NHW, Non-Hispanic White.

The age-adjusted prevalence of obesity in AoU participants varied from 59% in NHB women to 13% in Asian women (Table 1). When compared to NHANES 2015–2016 data, AoU participants had significantly lower age-adjusted obesity prevalence in NHW and NHB men and in NHW women, with AoU Asian men having a significantly higher rate of obesity (Fig 2 and S2 Table in S1 File). When compared to NHANES 2017–2018 data, AoU participants had significantly lower age-adjusted obesity prevalence in NHW and NHB men and in NHW women, with AoU Hispanic women having a higher rate of obesity. Notably, significant differences in age-adjusted obesity prevalence were also observed between NHANES 2015–2016 and NHANES 2017–2018 in NHW and Asian men (Fig 2 and S2 Table in S1 File).

The pseudo weight analysis indicated that AoU participants were over-represented with respect to proportion of women and NHW participants, while under-represented with respect to Hispanic participants relative to NHANES (S3 Table in S1 File) The AoU cohort also had a higher age relative to NHANES, with race/ethnicity and gender-adjusted age estimates being lower than unadjusted AoU values. Consistent results were observed in the AoU cohorts with and without ALT data. However, the age discrepancy between AoU and NHANES was more pronounced in the AoU cohort with ALT data and, importantly, obesity prevalence was higher in the AoU cohort with ALT data. These results were consistent with direct statistical comparisons of demographic and anthropometric variables between the subsets of AoU participants with and without measured ALT (S4 Table in S1 File). The AoU ALT cohort had significantly higher age and BMI with a greater proportion of women and NHW participants, and a lower proportion of NHB and Asian participants.

**Fig 3:**
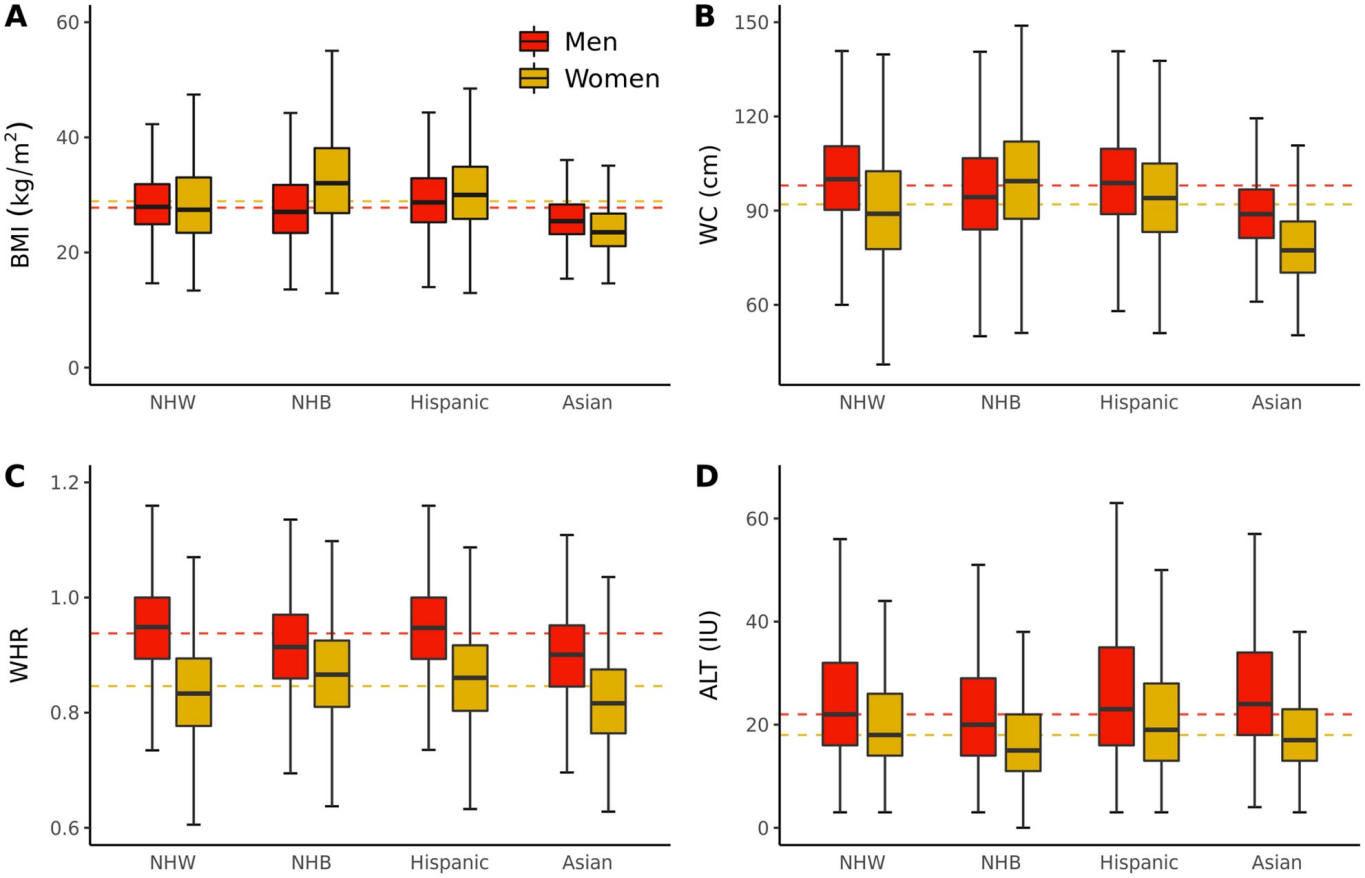
Anthropometric variables and ALT in all of us research program participants by gender and race/ethnicity. A) Body Mass Index (BMI), B) Waist Circumference (WC), C) Waist Hip Ratio (WHR), D) Alanine Aminotransferase (ALT). Horizontal dotted lines indicate the median body metric by gender. The boxplots visualize five summary statistics: Black lines indicate the median, lower and upper hinges reflect the 25th to 75th percentile, respectively, and the upper and lower error bars indicate the 5th and 95th percentiles, respectively. Due to privacy restrictions related to AoU publications policy, outlying observations are not plotted on boxplots. Men and women were included if they self-reported a single race/ethnic group in Asian, Black or African American, Hispanic or Latino, or White. NHB indicates Non-Hispanic Black and NHW, Non-Hispanic White.

Men exhibited higher levels of ALT than women in all racial/ethnic groups after exclusion of heavy drinkers. NHW, NHB, and Asian women exhibited lower levels of ALT compared to all three other gender/race/ethnicity groups (Table 1 and Fig 3). Multi-variable regressions indicated increasing ALT with increasing age (β = 0.20[0.05], p = 9.24x10^-6^), with increasing BMI (β = 0.21 [0.02], p = 2.08x10^-42^), and in Hispanic versus NHW participants (β = 1.39 [0.31], p = 5.97x10^-6^) (Table 3). A decrease in ALT was observed in women versus men (β = -6.07[0.25], p = 5.17x10^-128^) and in NHB versus NHW participants (β = -5.24 [0.32], p = 8.41x10^-60^). Significant race/ethnicity by gender interaction terms were also observed for NHB and Asian race/ethnicity groups (Table 3). In AoU, ALT was measured 90.3 days on average prior to measurement of BMI. The date of ALT measurement ranged from 4.5 years prior to BMI to 2.1 years after BMI.

**Table 3:** Results of multivariable linear regression models for ALT.

Covariate	β^a^	SE^a^	L95^a^	U95^a^	P Value^a^
**Multivariable Regression for ALT (R^2^ = 0.039)**					
Age (years)	0.20	0.05	0.11	0.29	9.24x10^-6^
Age^2^ (years)	-0.0031	0.0004	-0.0040	-0.0023	3.380x10^-13^
BMI	0.21	0.02	0.18	0.24	2.08x10^-42^
Women	-6.07	0.25	-6.56	-5.57	5.17x10^-128^
NHB	-5.24	0.32	-5.86	-4.61	8.41x10^-60^
Hispanic	1.39	0.31	0.79	2.00	5.97x10^-6^
Asian	-0.39	0.79	-1.94	1.15	0.617
Multivariable Regression for ALT with Race/Ethnicity*Gender Interaction Terms (R^2^ = 0.040)					
Age (years)	0.20	0.05	0.11	0.29	1.45x10^-5^
Age^2^ (years)	-0.0031	0.0004	-0.0039	-0.0022	7.76x10^-13^
BMI	0.21	0.02	0.18	0.24	3.45x10^-43^
Women	-5.57	0.32	-6.20	-4.93	1.14x10^-65^
NHB	-4.07	0.55	-5.16	-2.98	2.29x10^-13^
Hispanic	1.71	0.55	0.64	2.78	1.75x10^-3^
Asian	2.04	1.31	-0.52	4.60	0.118
Women*NHB	-1.73	0.67	-3.05	-0.42	9.93x10^-3^
Women*Hispanic	-0.48	0.65	-1.75	0.79	0.460
Women*Asian	-3.77	1.63	-6.97	-0.58	0.0021

ALT, alanine aminotransferase; BMI, body mass index; L95, lower limit of 95% confidence interval for beta; NHB, Non-Hispanic Black; NHW, Non-Hispanic White; SE, standard error; U95, upper limit of 95% confidence interval for beta.

^a^P values, betas, and standard errors, L95, and U95 for betas were generated using linear regressions with BMI as the outcome and age, age^2^, BMI, gender, and race/ethnicity as covariates.

In all regressions, men and NHW race were used as reference groups. Multiple linear regressions were performed with and without race/ethnicity*gender interaction terms. Significance was determined at an alpha level of 0.05. A normal distribution was assumed based on the large numbers of participants included for each subgroup. P values lower than 2.22x10^-308^

## Discussion

In this manuscript, we sought to evaluate racial/ethnic and gender disparities in obesity and body fat distribution in the first tranche of data from AoU. We observed strong disparities consistent with previously published literature, including the large gender discrepancy in obesity prevalence between NHB women and NHB men. Data from NHANES has shown that the prevalence of obesity is highest among NHB and Hispanic participants, and lower among NHW and Asian participants [5]. In NHANES, similar to our findings, gender differences in obesity are very pronounced in NHB individuals, in which the prevalence of obesity among NHB women is much higher than among NHB men [5], a pattern that has been attributed to genetic factors [16], as well as social and economic factors [17–20]. Our findings also replicate previous identified racial differences that exist with respect to body fat distribution. Data from NHANES has shown the prevalence of abdominal obesity to be highest for NHB women, followed by Mexican American women, and then NHW women [21]. Furthermore, the prevalence of non-alcoholic fatty liver disease differs strongly by racial/ethnic group, being highest in Hispanic Americans, and lowest in NHB individuals [7, 22]. In addition, we replicate the observation that NHB women have lower ALT despite a higher BMI, potentially suggesting a more “metabolically healthy” obesity phenotype in NHB women [23].

Our data suggest that AoU participants are generally representative of the US with respect to obesity prevalence. However, we do observe a consistently lower prevalence of obesity in AoU for NHW men and women and for NHB men compared to both NHANES datasets. This is an important consideration for future research in AoU, as healthier participants are often more likely to participate in cohort studies, such as the UK Biobank, resulting in a cohort that is not reflective of national averages [24]. It will be important to consider how participation in a non-probability sample such as the AoU could be influenced by a variety of other factors such as age, gender, socioeconomic status, and other factors at the social and individual levels. For example, we observed a greater proportion of women, as well as older and NHW participants in AoU compared to NHANES participants, especially in the AoU subset with ALT data. Future research conducted with AoU data will benefit from a consideration of the extent to which findings in AoU may or may not be generalizable to the rest of the US population.

The large sample size and racial/ethnic diversity of the cohort, combined with participant-provided questionnaires, retrospective and prospective EHR data, and standardized baseline measurements are some of the strengths that will set AoU apart from other large studies such as NHANES. The eventual availability of whole genome sequence data, biological specimens on all participants, and the ability to re-contact participants for research is also likely to bolster AoU’s potential scientific utility. These all point to AoU becoming a very important resource for future work in racial/ethnic disparities, as well as many other areas of biomedical research.

A number of limitations are worthy of mention in our study. As mentioned above, the AoU participants may not be reflective of the broader US population, and the recruitment approach could result in various biases and invalid inferences that will need to be considered in future research [15, 25, 26]. We did not account for comorbid disease or drug treatments that might affect obesity prevalence and ALT levels. Finally, the AoU Registered Tier was made up of a patchwork of datasets, creating inconsistency in availability of different types of data on individual participants. While anthropomorphic data was available on the vast majority of participants, only a subset of these individuals had PPI data, and a still smaller subset of patients had EHR data. A large variability in the amount of EHR data per participant was also observed. Consideration of the availability of this data is important, especially when defining criteria for cases and controls and in distinguishing what data elements are informative based on their absence or uninformativeness because of missingness.

## Conclusions

In this manuscript, we evaluated racial/ethnic and gender disparities in obesity and body fat distribution in newly emerging data from AoU. We replicated a number of known racial/ethnic and gender disparities related to obesity as well as ALT, a surrogate marker of hepatic steatosis. Our data suggest the consistency of the All of Us cohort with national averages related to obesity and indicate that this resource is likely to be a major source of scientific inquiry and discovery for decades to come, with an especially important contribution to understanding the genetic basis of disease in diverse populations.

## Supporting information

S1 File Supplementary materials. Supplementary methods and S1-S4 Tables.
(DOCX)

S2 File AoU basics survey (English). (PDF)

S3 File AoU basics survey (Spanish). (PDF)

S4 File AoU lifestyle survey (English). (PDF)

S5 File AoU lifestyle survey (Spanish). (PDF)
