# Supplementary material for: Racial, ethnic, and gender differences in obesity and body fat distribution: An All of Us Research Program demonstration project
Source: PLoS One. 2021 Aug 6;16(8):e0255583. doi: 10.1371/journal.pone.0255583 (PMC8345840; doi:10.1371/journal.pone.0255583)
Supplement: S1 File — Supplementary methods and S1-S4 Tables. (DOCX) [file pone.0255583.s001.docx]

**S1 File**

**Racial, Ethnic, and Gender Differences in Obesity and Body Fat Distribution: an All of Us Research Program Demonstration Project**

Jason H Karnes^1,2^, Amit Arora^3^, Jianglin Feng^1^, Heidi E Steiner^1^, Lina Sulieman^4^, Eric Boerwinkle^5^, Mine Cicek^6^, Cheryl Clark^7^, Elizabeth Cohn^8^, Kelly Gebo^9,10^, Roxana Loperena^4^, Kelsey Mayo^4^, Steve Mockrin^9^, Lucila Ohno-Machado^11^, Andrea Ramirez^12^, Sheri Schully^9^, Yann C Klimentidis ^3,13*^, on behalf of the All of Us Research Program Investigators^

^1^ Department of Pharmacy Practice and Science, College of Pharmacy, University of Arizona, Tucson, AZ, USA

^2^ Department of Biomedical Informatics, Vanderbilt University Medical Center, Nashville, TN, USA

^3^ Department of Epidemiology and Biostatistics, Mel and Enid Zuckerman College of Public Health, University of Arizona, Tucson, AZ, USA

^4^ Vanderbilt Institute for Clinical and Translational Research, Vanderbilt University Medical Center, Nashville, TN, USA

^5^ University of Texas Health Science Center at Houston, Houston, TX, USA

^6^ Department of Laboratory Medicine and Pathology, Mayo Clinic, Rochester, MN, USA

^7^ Department of Medicine, Brigham and Women's Hospital, Boston, MA, USA

^8^ Hunter-Bellevue School of Nursing, Hunter College, City University of New York, New York, NY, USA

^9^ All of Us Research Program, National Institutes of Health, Bethesda, MA, USA

^10^ Johns Hopkins University School of Medicine, Baltimore, MD

^11^ Department of Biomedical Informatics, University of California San Diego Health, San Diego, CA, USA

^12^ Department of Medicine, Vanderbilt University Medical Center, Nashville, TN, USA

^13^ BIO5 Institute, University of Arizona, Tucson, AZ, USA

^*^ Address Correspondence to:

[yann@email.arizona.edu](mailto:yann@email.arizona.edu) (YCK)

^Membership of the All of Us Research Program Investigators is provided in the Acknowledgements.

**Supplemental Methods**

All of Us Research Program (AoU) Design

We used data from the AoU Researcher Workbench, which houses data for physical measurements, PPI, and EHRs. When EHR data was available for a consented participant, it was provided to the AoU Data and Research Center (DRC) by individual recruitment sites. EHR data was subsequently de-identified and stored in structured format in the Research Workbench using the Observational Medical Outcomes Partnership (OMOP) Common Data Model (12). AoU Demonstration Projects were granted early access to Registered Tier AOU cohort data in December 2019 with purpose to use Researcher Workbench tools to characterize and validate the AoU cohort and data in order to demonstrate the quality, utility, and diversity of AoU data and tools. Registered Tier data underwent privacy methodology to remove explicit identifiers, free-text fields (PPI), full-text documents (EHR), geo-location data smaller than US state, living situation (PPI), active duty military status (PPI), cause of death (EHR), and race and ethnicity subgroups (PPI). Registered Tier data also underwent data generalization process to ensure participant privacy, which included removal of all participants age 89 and above as well as under-represented race/ethnic group, gender identity, sexual orientation, and educational attainment classifications.

Anthropometric Measurements

A standardized set of physical measurements were obtained from participants at the baseline visit. Measurements were recorded by a trained program staff member in HealthPro, the dedicated AoU platform for logging results of physical measurements, processing biospecimen collections, and viewing individual-level participant operational data.

*Height and Weight:* All height and weight measurements were obtained on the participant without shoes and without bulky clothing. Height was taken with the participant standing erect on the stadiometer platform/floor with his/her back against the vertical-mounted centimeter ruler, heels against the wall, and feet or knees together, whichever come together first. The participant’s head was aligned such that the horizontal line from the ear canal to the lower border of the orbit of the eye is parallel to the floor and perpendicular to the vertical backboard. After the stadiometer head piece was rested firmly on top of the participant’s head, the participant was instructed to stand as tall as possible, take a deep breath, and hold this position. Modifications to the height measurement were recorded and allowed if participants were wheelchair users, taller than the stadiometer, or had impediments such as head gear or hairstyle. Weight was taken using a well-maintained scale (calibrated at least once a year) with a capacity of at least 440 pounds and an ability to determine weight in kilograms rounded to a tenth of a kilogram. The scale was placed on level and firm ground and balanced so that the scale indicator was at zero when no weight is on the scale and the participant was instructed to stand in the middle of the platform with head erect and eyes looking straight ahead. Modifications to the weight measurement were recorded and allowed if participants weigh more than the measuring range of the scale (self-reported weight or maximum scale weight was used), the participant was a wheelchair user (self-reported weight was used), or the participant was pregnant (self-reported pre-pregnancy weight was used).

*Waist and Hip Circumference:* Waist and hip circumference were not measured if a participant was pregnant, a wheelchair user, wearing a colostomy bag, or was not willing to be measured. Briefly, participants were asked to remove bulky items or clothing, to stand with feet positioned close together and weight evenly distributed across both feet, and to relax and take a few deep breaths. Waist circumference was measured at the end of normal expiration using a non-stretchable tape measure (rounded to the nearest millimeter) at the smallest part of the trunk, the top of the hip bones, or the umbilicus. Two consecutive measurements were obtained with a third measurement taken if the first two measurements differed by >1.0 cm. If a third measurement was performed, the two closest measurements were automatically averaged unless the third measurement fell equally between the first two, in which case all three measurements were averaged.

EHR Laboratory Results: Alanine Aminotransferase

Levels of alanine aminotransferase (ALT) were obtained from the EHR records of participants. We used the lab measurement “Alanine aminotransferase [Enzymatic activity/volume] in Serum or Plasma” with the Logical Observation Identifiers Names and Codes (LOINC) code 1742-6. This LOINC code constituted the ALT measurement for which the greatest number of individuals had data. If more than one ALT measurement was available for a given participant, the most recent lab measurement was used in our analyses. Only ALT measurements acquired in 2015 and afterwards we used in this analysis and the most recent ALT measurement was used if multiple measurements were available on a participant. Participants were excluded from ALT analyses if multiple ALT measures were available at the same date and time but with a different ALT result.

**Supplementary Table S1:** Comparison of anthropometric measures and ALT between men and women within each racial/ethnic group.

| **Race/**  **Ethnicity** | **Mean (men)** | **Mean (women)** | **t^a^** | **L95^a^** | **U95^a^** | **P Value^a^** |
| --- | --- | --- | --- | --- | --- | --- |
| **Body Mass Index (kg/m^2^)** | | | | | | |
| NHW | 29.02 | 29.09 | -1.55 | -0.17 | 0.02 | 0.12 |
| NHB | 28.40 | 33.34 | -60.63 | -5.09 | -4.77 | <2.22x10^-308^ |
| Hispanic | 29.75 | 31.06 | -16.68 | -1.46 | -1.15 | 3.60x10^-62^ |
| Asian | 26.12 | 24.49 | 12.56 | 1.38 | 1.89 | 1.18x10^-35^ |
| **Waist to Hip Ratio** | | | | | | |
| NHW | 0.95 | 0.84 | 162.06 | 0.109 | 0.112 | <2.22x10^-308^ |
| NHB | 0.92 | 0.87 | 45.70 | 0.047 | 0.051 | <2.22x10^-308^ |
| Hispanic | 0.95 | 0.86 | 75.69 | 0.08 | 0.09 | <2.22x10^-308^ |
| Asian | 0.90 | 0.82 | 29.68 | 0.07 | 0.09 | 7.04x10^-174^ |
| **Waist Circumference (cm)** | | | | | | |
| NHW | 101.28 | 91.34 | 84.06 | 9.71 | 10.18 | <2.22x10^-308^ |
| NHB | 96.94 | 100.47 | -19.05 | -3.89 | -3.17 | 1.55x10^-80^ |
| Hispanic | 100.15 | 94.83 | 27.66 | 4.94 | 5.70 | 1.11x10^-165^ |
| Asian | 89.70 | 79.30 | 29.28 | 9.71 | 11.10 | 1.22x10^-172^ |
| **ALT Level (IU/L)** | | | | | | |
| NHW | 27.92 | 22.92 | 15.88 | 4.38 | 5.62 | 3.24x10^-56^ |
| NHB | 25.33 | 18.82 | 12.48 | 5.49 | 7.53 | 6.04x10^-35^ |
| Hispanic | 31.07 | 25.75 | 7.61 | 3.94 | 6.68 | 3.54x10^-14^ |
| Asian | 30.52 | 21.08 | 5.62 | 6.14 | 12.75 | 3.26x10^-8^ |

ALT indicates alanine aminotransferase; cm, centimeters; IU/L, international units; L95, lower limit of 95% confidence interval for mean; NHB, Non-Hispanic Black; NHW, Non-Hispanic White; U95, upper limit of 95% confidence interval for mean; WHR, waist-to-hip ratio.

^a^t statistic, L95, U95, and p values were generated using Student’s t-tests. Significance was determined at an alpha level of 0.05. A normal distribution was assumed based on the large numbers of participants included for each subgroup. P values lower than 2.22x10^-308^ were not calculated.

**Supplementary Table S2:** Comparison of age-adjusted obesity prevalence in NHANES 2015-2016, NHANES 2017-2018, and AllofUs.

| **Race/Ethnicity and Gender Category** | **Obesity in**  **NHANES**  **2015-16,**  **% (L95-U95)^a^** | **Obesity in**  **NHANES 2017-18,**  **% (L95-U95)^a^** | **Obesity in**  **AllofUs, % (L95-U95)^a^** | **P Value (NHANES 2015-16 vs. 2017-18)^b^** | **P Value (NHANES 2015-16 vs. AllofUs)^b^** | **P Value (NHANES 2017-18 vs. AllofUs)^b^** |
| --- | --- | --- | --- | --- | --- | --- |
| NHW Women | 40.5 (37.1, 43.9) | 43.2 (39.6, 46.8) | 36.0 (35.5, 36.5) | 0.29 | 0.01 | 1.14x10^-4^ |
| NHW Men | 37.7 (34.3, 41.2) | 44.5 (40.7, 48.3) | 32.8 (32.1, 33.4) | 0.01 | 0.006 | 3.67x10^-9^ |
| NHB Women | 55.7 (51.5, 60.0) | 57.3 (52.7, 61.8) | 58.7 (57.9, 59.5) | 0.62 | 0.17 | 0.53 |
| NHB Men | 37.1 (32.5, 41.7) | 39.5 (34.5, 44.6) | 32.0 (31.0, 33.0) | 0.49 | 0.03 | 0.004 |
| Hispanic Women | 48.3 (44.7, 51.9) | 43.0 (38.5, 47.6) | 49.2 (48.4, 49.9) | 0.08 | 0.66 | 0.01 |
| Hispanic Men | 42.4 (38.3, 46.5) | 45.0 (40.1, 50.0) | 40.1 (39.0, 41.2) | 0.43 | 0.28 | 0.06 |
| Asian Women | 13.6 (9.5, 17.8) | 16.9 (12.5, 21.3) | 12.8 (11.5, 14.0) | 0.29 | 0.70 | 0.08 |
| Asian Men | 9.7 (6.3, 13.2) | 16.8 (12.3, 21.3) | 16.6 (14.9, 18.3) | 0.02 | 5.25x10^-4^ | 0.95 |

ALT indicates alanine aminotransferase; BMI, body mass index; L95, lower limit of 95% confidence interval for mean; NHANES, National Health and Nutrition Examination Survey; NHB, Non-Hispanic Black; NHW, Non-Hispanic White; U95, upper limit of 95% confidence interval for mean.

^a^Age-adjusted obesity prevalence was calculated by matching the distribution to that of the 2010 US Census using six age groups (18-29, 30-39, 40-49, 50-59, 60-69, and 70 and over). For each gender and race/ethnicity subset, we use proportions of the six age groups in the US population for each subset as reference weights to obtain the weights for AoU and NHANES cohorts, to calculate the weighted mean, and to calculate the variance where Kish’s effective sample size is used for calculated the confidence interval.

^b^P Values were generated using Student’s t-tests. Significance was determined at an alpha level of 0.05. A normal distribution was assumed based on the large numbers of participants included for each subgroup.

**Supplementary Table S3:** Bias-adjustment of AoU variables with NHANES as reference sample by using pseudo weighting method. Age, gender, and race/ethnicity adjusted means of variables in two AoU samples are presented.

| **Variable** | **NHANES^a^**  **(std error)** | **AoU^b^**  **(n=136,395)** | | **AoU with ALT^b^ (n=30,485)** | |
| --- | --- | --- | --- | --- | --- |
|  |  | **Un-adjusted** | **Adjusted^c^** | **Un-adjusted** | **Adjusted^c^** |
| Women (%) | 51.4 (0.5) | 60.1 | 51.5 | 65.8 | 51.6 |
| Hispanic (%) | 28.8 (0.5) | 20.7 | 28.8 | 20.2 | 29.4 |
| NHW (%) | 35.8 (0.5) | 52.8 | 35.7 | 58.5 | 35.1 |
| NHB (%) | 22.2 (0.4) | 23.1 | 22.1 | 19.0 | 21.8 |
| Age (years) | 48.7 (0.2) | 51.5 | 48.6 | 55.2 | 48.6 |
| Height (cm) | 166.4 (0.1) | 167.8 | 168.0 | 166.7 | 167.8 |
| Weight (kg) | 81.8 (0.2) | 83.5 | 82.3 | 85.3 | 84.6 |
| BMI (kg/m^2^) | 29.4 (0.1) | 29.7 | 29.1 | 30.7 | 30.0 |
| WC (cm) | 99.9 (0.2) | 96.0 | 94.8 | 98.6 | 97.4 |
| Obesity (%) | 40.1 (0.1) | 40.1 | 38.1 | 46.1 | 41.9 |
| ALT (IU/L) | 23.9 (0.2) | -- | -- | 23.7 | 24.8 |

ALT indicates alanine aminotransferase; BMI, body mass index; cm, centimeters; IU/L, international units per liter; NHANES, National Health and Nutrition Examination Survey; NHB, Non-Hispanic Black; NHW, Non-Hispanic White; WC, waist circumference.

^a^ The NHANES reference sample were generated by combining NHANES 2015-2016 and NHANES 2017-2018 samples. Individuals with unknown gender, unknown race/ethnicity or age less than 18 are removed to match those restrictions of AoU and *AoU with ALT* samples.

^b^ AoU sample were generated from the subset of AoU data from 2015-2018. The *AoU with ALT* sample were further restricted to only participants that had ALT from their electronic health record.

^c^ Age, gender, and race/ethnicity adjusted means were calculated using pseudo-weight method. The first 5 variables in the table plus the quadratic term of age are used to obtain the pseudo weights by using logistic regression on the combined sample (NHANES sample and the general AoU sample, or NHANES sample and the *AoU with ALT* sample), and these weights are then used to estimate the means of other variables (height, weight, BMI, WC, obesity and ALT). Since the reference NHANES sample is not a strict probability sample, the adjusted means of AoU variables are only relevant to NHANES sample.

**Supplementary Table S4:** Comparison of demographic and anthropometric variables between subsets of AllofUs participants with measured ALT and the subset of AllofUs participants with no measured ALT.

| **Variable** | **AllofUs with ALT**  **(n=33,772)** | **AllofUs with no ALT**  **(n=136,481)** | **P Value^a^** |
| --- | --- | --- | --- |
| Age (mean [SD]) | 55.26 (15.89) | 51.00 (16.61) | <2.22x10^-308^ |
| BMI (mean [SD]) | 30.88 (7.93) | 29.48 (7.46) | 4.38x10^-183^ |
| Women (n [%]) | 22458 (66.4) | 80485 (58.9) | 1.43x10^-141^ |
| NHW (n [%]) | 19420 (57.5) | 68775 (50.3) | 2.80x10^-121^ |
| NHB (n [%]) | 6348 (18.7) | 34422 (25.2) | 2.80x10^-121^ |
| Hispanic (n [%]) | 7226 (21.3) | 28414 (20.8) | 0.002 |
| Asian (n [%]) | 778 (2.3) | 4870 (3.5) | 3.33x10^-31^ |

ALT indicates alanine aminotransferase; BMI, body mass index; NHB, Non-Hispanic Black; NHW, Non-Hispanic White.

^a^P Values were generated using Student’s t-tests. Significance was determined at an alpha level of 0.05. A normal distribution was assumed based on the large numbers of participants included for each subgroup. P values lower than 2.22x10^-308^ were not calculated.
