# Supplementary material for: Racial, ethnic, and gender differences in obesity and body fat distribution: An All of Us Research Program demonstration project
Source: PLoS One. 2021 Aug 6;16(8):e0255583. doi: 10.1371/journal.pone.0255583 (PMC8345840; doi:10.1371/journal.pone.0255583)
Supplement: S2 File — (PDF) [file pone.0255583.s002.pdf]

The Basics

This survey asks questions about you, your work, and your home life. This is to better understand how they may affect health. To ensure your privacy, your name will be separated from your answers before they are shared with researchers.

It takes about 10-15 minutes to answer these questions. Please answer each question as honestly as possible. There are no right or wrong answers to any of the questions. It is important that you answer as many questions as you can. We are looking for your own answers, and not what you think your doctors, family, or friends want you to say.

Don't feel like you have to spend a long time on each question. The first answer that comes to you is usually the best one. If you aren't sure how to answer a question, choose the best answer from the options given.

The first 9 questions ask about basic background information.

In what country were you born?[1]

- USA
- Other (free text)

Which categories describe you? Select all that apply. Note, you may select more than one group.[2]

- ❑ American Indian or Alaska Native (For example: Aztec, Blackfeet Tribe, Mayan, Navajo Nation, Native Village of Barrow (Utqiagvik) Inupiat Traditional Government, Nome Eskimo Community, etc.)
  Branching Logic: when "American Indian or Alaska Native" selected, then:
  - ❑ American Indian
    Branching Logic: when "American Indian" selected, then:
    Provide the name of the tribe in which you are enrolled or affiliated or your tribal descent (For example: Aztec, Blackfeet Tribe, Mayan, Navajo Nation, Native Village of Barrow (Utqiagvik) Inupiat Traditional Government, Nome Eskimo Community, etc.)
    Please specify.[2]
      - (*display optional free text*)
  - ❑ Alaska Native
    Branching Logic: if "Alaska Native" selected, then:
    Provide the name of the tribe in which you are enrolled or affiliated or your tribal descent (For example: Aztec, Blackfeet Tribe, Mayan, Navajo Nation, Native Village of Barrow (Utqiagvik) Inupiat Traditional Government, Nome Eskimo Community, etc.)
    Please specify.[2]

- (*display optional free text*)

- ❑ Central or South American Indian
  Branching Logic: when "Central or South American Indian" selected, then:
  Provide the name of the tribe in which you are enrolled or affiliated or your tribal descent (For example: Aztec, Blackfeet Tribe, Mayan, Navajo Nation, Native Village of Barrow (Utqiagvik) Inupiat Traditional Government, Nome Eskimo Community, etc.)
  Please specify.[2]
  - (*display optional free text*)
- ❑ None of these fully describe me
  Branching Logic: when "None of these fully describe me" selected, then:
  Please specify.[2]
  - (*display optional free text*)

- Asian (For example: Asian Indian, Chinese, Filipino, Japanese, Korean, Vietnamese, etc.)
  Branching Logic: when "Asian" selected, then select:
  - ❑ Asian Indian
  - ❑ Cambodian
  - ❑ Chinese
  - ❑ Filipino
  - ❑ Hmong
  - ❑ Japanese
  - ❑ Korean
  - ❑ Pakistani
  - ❑ Vietnamese
  - ❑ None of these fully describe me
    Branching Logic: when "None of these fully describe me" selected, then:
    Please specify.[2]
    - (*display optional free text*)

- ❑ Black, African American, or African (For example: African American, Ethiopian, Haitian, Jamaican, Nigerian, Somali, etc.)
  Branching Logic: when "Black, African American, or African" selected, then select:
  - ❑ African American
  - ❑ Barbadian
  - ❑ Caribbean
  - ❑ Ethiopian
  - ❑ Ghanaian
  - ❑ Haitian
  - ❑ Jamaican
  - ❑ Liberian
  - ❑ Nigerian
  - ❑ Somali
  - ❑ South African

- ❑ None of these fully describe me
  Branching Logic: when "None of these fully describe me" selected, then:
  Please specify.[2]
    - (*display optional free text*)

❑ Hispanic, Latino, or Spanish (For example: Colombian, Cuban, Dominican, Mexican or Mexican American, Puerto Rican, Salvadoran, etc.)
Branching Logic: when "Hispanic, Latino, or Spanish" selected, then select:
- ❑ Colombian
- ❑ Cuban
- ❑ Dominican
- ❑ Ecuadorian
- ❑ Honduran
- ❑ Mexican or Mexican American
- ❑ Puerto Rican
- ❑ Salvadoran
- ❑ Spanish
- ❑ None of these fully describe me
  Branching Logic: when "None of these fully describe me" selected, then:
  Please specify.[2]
    - (*display optional free text*)

- Middle Eastern or North African (For example: Algerian, Egyptian, Iranian, Lebanese, Moroccan, Syrian, etc.)
  Branching Logic: when "Middle Eastern or North African" selected, then select:
    - ❑ Afghan
    - ❑ Algerian
    - ❑ Egyptian
    - ❑ Iranian
    - ❑ Iraqi
    - ❑ Israeli
    - ❑ Lebanese
    - ❑ Moroccan
    - ❑ Syrian
    - ❑ Tunisian
    - ❑ None of these fully describe me
      Branching Logic: when "None of these fully describe me" selected, then:
      Please specify.[2]
        - (*display optional free text*)

- Native Hawaiian or other Pacific Islander (For example: Chamorro, Fijian, Marshallese, Native Hawaiian, Tongan, etc.)
  Branching Logic: when "Native Hawaiian or other Pacific Islander" selected, then select:
    - ❑ Chamorro

- ❑ Chuukese
- ❑ Fijian
- ❑ Marshallese
- ❑ Native Hawaiian
- ❑ Palauan
- ❑ Samoan
- ❑ Tahitian
- ❑ Tongan
- ❑ None of these fully describe me

  Branching Logic: when "None of these fully describe me" selected, then:

  Please specify.[2]
  - (*display optional free text*)

- White (For example: English, European, French, German, Irish, Italian, Polish, etc.)

  Branching Logic: when "White" selected, then select:
  - ❑ Dutch
  - ❑ English
  - ❑ European
  - ❑ French
  - ❑ German
  - ❑ Irish
  - ❑ Italian
  - ❑ Norwegian
  - ❑ Polish
  - ❑ Scottish
  - ❑ Spanish
  - ❑ None of these fully describe me

    Branching Logic: when "None of these fully describe me" selected, then:

    Please specify.[2]
    - (*display optional free text*)

- ❑ None of these fully describe me

  Branching Logic: when "None of these fully describe me" selected, then:

  Please specify.[2]
  - (*display optional free text*)
- ❑ Prefer not to answer

What terms best express how you describe your gender identity? (Check all that apply)[3]

- ❑ Man
- ❑ Woman
- ❑ Non-binary
- ❑ Transgender
- ❑ None of these describe me, and I'd like to consider additional options
- ❑ Prefer not to answer

Branching Logic: when "non-binary," "transgender," or "None of these describe me, and I'd like to consider additional options" selected, then:

Are any of these a closer description to your gender identity? (Check all that apply)[3]

- ❑ Trans man/Transgender Man/FTM
- ❑ Trans woman/Transgender Woman/MTF
- ❑ Genderqueer
- ❑ Genderfluid
- ❑ Gender variant
- ❑ Two-spirit
- ❑ Questioning or unsure of your gender identity
- ❑ None of these describe me, and I want to specify

  Branching Logic: when "None of these fully describe me, and I want to specify" selected, then:

  Please specify.[2]

  (display optional free text)

What was your biological sex assigned at birth?[3]

- Female
- Male
- Intersex
- None of these describe me

  Branching Logic: when "None of these fully describe me" selected, then:

  Please specify.[3]

  - *(display optional free text)*
- Prefer not to answer

Which of the following best represents how you think of yourself? (Check all that apply)[4]

- ❑ Gay
- ❑ Lesbian
- ❑ Straight; that is, not gay or lesbian, etc.
- ❑ Bisexual
- ❑ None of these describe me, and I'd like to see additional options

  Branching Logic: when "none of these describe me, and I'd like to see additional options" selected, then:

  Are any of these a closer description of how you think of yourself?[5]

  - ○ Queer
  - ○ Polysexual, omnisexual, sapiosexual or pansexual
  - ○ Asexual
  - ○ Two-spirit
  - ○ Have not figured out or are in the process of figuring out your sexuality
  - ○ Mostly straight, but sometimes attracted to people of your own sex

- ○ Do not think of yourself as having sexuality
- ○ Do not use labels to identify yourself
- ○ Don't know the answer
- ○ No, I mean something else

  Branching Logic: when "No, I mean something else" selected, then:

  Please specify.[5]

  - (*display optional free text*)

❏ Prefer not to answer

What is the highest grade or year of school you completed?[5]

- Never attended school or only attended kindergarten
- Grades 1 through 4 (Primary)
- Grades 5 through 8 (Middle school)
- Grades 9 through 11 (Some high school)
- Grade 12 or GED (High school graduate)
- 1 to 3 years after high school (Some college, Associate's degree, or technical school)
- College 4 years or more (College graduate)
- Advanced degree (Master's, Doctorate, etc.)
- Prefer not to answer

Have you ever served on active duty in the United States Armed forces, either in the regular military or in a National Guard or military reserve unit?

Note: Active duty does not include training for the Reserves or National Guard, but DOES include activation, for example, for the Persian Gulf War[5]

- Yes
- No
- Prefer not to answer

What is your current marital status?[5]

- Married
- Divorced
- Widowed
- Separated
- Never married
- Living with partner
- Prefer not to answer

The next 2 questions ask about any people who live with you.

Not including yourself, how many other people live at home with you? [5]

- Free text (Integer value)

  Branching logic: when any number other than "0" is entered, then:
  Think of other people who live with you. How many are under the age of 18 years?[1]
  - Free text (Integer value)

The next questions are about health insurance. Include health insurance obtained through employment or purchased directly as well as government programs like Medicare and Medicaid that provide medical care or help pay medical bills.

Are you covered by health insurance or some other kind of health care plan?[1]

- Yes
  Branching logic: when "Yes" selected, then:
  Are you covered by health insurance or some other kind of health care plan?[1]
  - Insurance purchased directly from an insurance company (by you or another family member)
  - Insurance through a current or former employer or union (by you or another family member)
  - Medicare, for people 65 and older or people with certain disabilities
  - Medicaid, Medical Assistance, or any kind of government-assistance plan for those with low incomes or disability
  - TRICARE or other military health care
  - Veterans Affairs (VA) (including those who have ever used or enrolled for VA health care)
  - Indian Health Service
  - Any other type of health insurance or health coverage plan
    Branching logic: when "Any other type of health insurance or health coverage plan" is selected, then:
    Please specify:[1]
    (free text)
  - I don' t have health insurance, self-pay
- No
- Don't know
- Prefer not to answer

The next questions ask if you have a disability. Some questions will ask you about more than one disability at a time. Please answer "Yes" if you have any one of them. *(info button text: In 1990, Congress passed a civil rights law to protect people with disabilities. The name of that law is the ADA (Americans with Disabilities Act). Having a disability means you might have a physical or mental problem. That problem might make it hard to do certain things. You might have a*

problem with: walking, breathing, learning, reading, communicating, seeing, hearing, or thinking.)

Are you deaf or do you have serious difficulty hearing?[10]

- Yes
- No
- Prefer not to answer

Are you blind or do you have serious difficulty seeing, even when wearing glasses?[10]

- Yes
- No
- Prefer not to answer

Because of a physical, mental, or emotional condition, do you have serious difficulty concentrating, remembering or making decisions?[10]

- Yes
- No
- Prefer not to answer

Do you have serious difficulty walking or climbing stairs?[10]

- Yes
- No
- Prefer not to answer

Do you have difficulty dressing or bathing?[10]

- Yes
- No
- Prefer not to answer

Because of a physical, mental, or emotional condition, do you have difficulty doing errands alone such as visiting doctor's office or shopping?[10]

- Yes
- No
- Prefer not to answer

The next questions are about your job, income, and where you live.

What is your current employment status? Please select 1 or more of these categories.[5]

- ❏ Employed for wages (part- time or full-time)
- ❏ Self-employed
- ❏ Out of work for 1 year or more
- ❏ Out of work for less than 1 year
- ❏ A homemaker
- ❏ A student

- ❏ Retired
- ❏ Unable to work (disabled)
- ❏ Prefer not to answer

Branching logic: when "employed for wages or self-employed" selected, then:
Sharing where you work may help us learn about how the environment affects health.

Sharing your work address is your choice. You can say no and still take part in the program.

What is your work street address?[10]

- ○ Enter Address
  Branching Logic: when "Enter Address" selected, then:
  Address Line 1[10]
  ____
  Address Line 2 (optional)[10]
  ____
  City[10]
  ____
  State[10]
  ____
  Zip code[10]
  ____
  Country[10]
  ____
- ○ Prefer not to answer

One of the things we're trying to understand is how people's income may affect their use of health services. Household income includes your income plus the income of all family members in your household for the last calendar year. Include all wages and other sources of income.

What is your annual household income from all sources?[5]

- Less than $10,000
- $10,000- $24,999
- $25,000-$34,999
- $35,000-$49,999
- $50,000- $74,999
- $75,000-$99,999
- $100,000- $149,999
- $150,000- $199,999
- $200,000 or more

- Prefer not to answer

Do you own or rent the place where you live?[7]

- Own
- Rent
- Other arrangement
  Branching logic: when "Other arrangement" selected, then:
  Where are you currently living?[7]
    - On a college campus
    - With a friend/roommate
    - With family
    - Motel/hotel
    - Hospital, rehabilitation center, drug treatment center, or other temporary institution
    - In a group home, nursing home, or other residential facility
    - Transitional housing
    - Emergency shelter or homeless shelter
    - Anywhere outside (e.g., street, vehicle, abandoned building)
    - Other
      Branching Logic: when "Other" selected, then:
      Please specify.[7]
      (free text)
- Prefer not to answer

How many years have you lived at your current address?[8]

- Less than 1 year
- 1-2 years
- 3-5 years
- 6-10 years
- 11-20 years
- More than 20 years

The next question is about stress that you may feel about money.

In the past 6 months, have you been worried or concerned about NOT having a place to live?[9]

- Yes
- No

If you have a Social Security Number, sharing it with us may help add extra data to the All of Us database. This extra data may come from places like your health care provider or pharmacy. Sharing your Social Security Number is your choice. You can say no and still take part in the program.

What is your Social Security Number?[6]

- Enter Social Security number
  Branching Logic: when "Enter Social Security Number" selected, then:
  Social Security Number[6]
  (social security number)
- Prefer not to answer

To help us stay in touch with you in the future, the last section asks for contact information for family and/or friends. This information is not required in order to participate in the program. All information will be securely stored.

The *All of Us* Research Program may contact you periodically to gather additional health related information. In case we cannot contact you, please provide the names, addresses, and telephone numbers of 2 relatives or friends who would know where you could be reached in case we have trouble reaching you. (Please give us the names of persons not currently living in the household)[1]

- **Person 1 First Name**[1]
  ____
- **Person 1 Middle Initial**[1]
  ____
- **Person 1 Last Name**[1]
  ____
- **Person 1 Address 1**[1]
  ____
- **Person 1 Address 2**[1]
  ____
- **Person 1 City**[1]
  ____
- **Person 1 State**[1]
  ____
- **Person 1 Zip Code**[1]
  ____
- **Person 1 Email Address**[1]
  ____
- **Person 1 Phone Number**[1] *(allow none, refused, or don't know)*
  ____
- **Relationship to You**[1]
  - Child
  - Friend
  - Parent or Guardian
  - Relative
  - Spouse or Partner

- **Person 2 First Name**[1]
  ____
- **Person 2 Middle Initial**[1]
  ____
- **Person 2 Last Name**[1]
  ____
- **Person 2 Address 1**[1]
  ____
- **Person 2 Address 2**[1]
  ____
- **Person 2 City**[1]
  ____
- **Person 2 State**[1]
  ____
- **Person 2 Zip Code**[1]
  ____
- **Person 2 Email Address**[1]
  ____
- **Person 2 Phone Number**[1] *(allow none, refused, or don't know)*
  ____
- **Relationship to You**[1]
  - Child
  - Friend
  - Parent or Guardian
  - Relative
  - Spouse or Partner

Thank you for completing the Basics survey.

The information you have shared may contribute to helping researchers improve the health of generations to come.

Sources

1. National Health and Nutrition Examination Survey (NHANES)
2. U.S. 2020 Census (2015 Draft)
3. GenIUSS group (Gender Identity in U.S. Surveillance)
4. National Health Interview Survey (NHIS)
5. Behavioral Risk Factor Surveillance System (BRFSS)
6. Developed for use in *All of Us*
7. National Health Care for the Homeless Council (NHCHC)
8. UK Biobank
9. VA Homelessness Screening Clinical Reminder (HSCR)
10. American Community Survey (ACS) (added to Basics October 2019)
