# Supplementary material for: Racial, ethnic, and gender differences in obesity and body fat distribution: An All of Us Research Program demonstration project
Source: PLoS One. 2021 Aug 6;16(8):e0255583. doi: 10.1371/journal.pone.0255583 (PMC8345840; doi:10.1371/journal.pone.0255583)
Supplement: S3 File — (PDF) [file pone.0255583.s003.pdf]

Lo Básico

Esta encuesta le hace preguntas sobre usted, su trabajo y su vida familiar. Esta información nos sirve para comprender mejor como estos aspectos pueden afectar su salud. Para asegurar su privacidad, su nombre se separará de sus respuestas antes de compartirlas con los investigadores.

Responder a las siguientes preguntas le tomará como 10-15 minutos. Por favor responda cada pregunta lo más honesto posible. Para cualquiera de estas preguntas no hay respuestas correctas o incorrectas. Es importante que responda a todas las preguntas que pueda. Estamos buscando sus propias respuestas, y no lo que piensan sus doctores, familiares, o amigos.

No sienta que tiene que tomar mucho tiempo para cada pregunta. La primera respuesta que se le ocurre es usualmente la mejor. Si no está seguro(a) como responder la pregunta, escoja la mejor respuesta de las opciones presentadas.

Las primeras 9 preguntas son sobre su información básica.

¿En qué país nació usted?[1]

- Estados Unidos
- Otro país
  Branching Logic: when "Otro país" selected, then:
  Por favor, especifique.[1]
  ____

¿Qué categorías lo o la describen? Marque todas las respuestas que correspondan. Nota: puede seleccionar más de un grupo. [2]

- ❑ Indio Americano o Nativo de Alaska (por ejemplo, Navajo Nation, Blackfeet Tribe, Maya, Azteca, Native Village of Barrow Inupiat, Nome Eskimo Community, etc)
  Branching Logic: when "Indio americano o nativo de Alaska" selected, then:
  - ❑ Indio americano
    Branching Logic: when "Indio americano" selected, then:
    Provea el nombre de la tribu en la cual usted está registrado o afiliado o su origen tribal (por ejemplo, Navajo Nation, Blackfeet Tribe, maya, azteca, Native Village of Barrow Inupiat, Nome Eskimo Community, etc.)[2]
    - *(texto libre)*
  - ❑ Nativo de Alaska
    Branching Logic: when "Nativo de Alaska" selected, then:

Provea el nombre de la tribu en la cual usted está registrado o afiliado o su origen tribal (por ejemplo, Navajo Nation, Blackfeet Tribe, maya, azteca, Native Village of Barrow Inupiat, Nome Eskimo Community, etc.)
Por favor, especifique.[2]
- *(texto libre)*

❑ Indio centroamericano o sudamericano
Branching Logic: when "Indio centroamericano o sudamericano" selected, then:
Provea el nombre de la tribu en la cual usted está registrado o afiliado o su origen tribal (por ejemplo, Navajo Nation, Blackfeet Tribe, maya, azteca, Native Village of Barrow Inupiat, Nome Eskimo Community, etc.)
Por favor, especifique.[2]
- *(texto libre)*

❑ Ninguna de estas me describe completamente
Branching Logic: when "Ninguna de estas me describe completamente" selected, then:
Por favor, especifique.[2]
- *(texto libre)*

❑ Asiático (por ejemplo, Chino, Filipino, Indio Asiático, Vietnamita, Coreano, Japonés, etc.)
Branching Logic: when "Asiático" selected, then:
❑ Indio asiático
❑ Camboyano
❑ Chino
❑ Filipino
❑ Hmong
❑ Japonés
❑ Coreano
❑ Pakistaní
❑ Vietnamita
❑ Ninguna de estas me describe completamente
Branching Logic: when "Ninguna de estas me describe completamente" selected, then:
Por favor, especifique.[2]
- *(texto libre)*

❑ Negro, Africano, o Afroamericano (por ejemplo, Africano Americano, Jamaiquino, Haitiano, Nigeriano, Etíope, Somalí, etc.)
Branching Logic: when "Negro o afroamericano" selected, then:
❑ Afroamericano
❑ Barbadeño
❑ Caribe
❑ Etíope
❑ Ghanés

- ❑ Haitiano
- ❑ Jamaiquino
- ❑ Liberiano
- ❑ Nigeriano
- ❑ Somalí
- ❑ Sudafricano
- ❑ Ninguna de estas me describe completamente

  Branching Logic: when "Ninguna de estas me describe completamente" selected, then:

  Por favor, especifique.[2]

  - *(texto libre)*

❑ Hispano, Latino o Español (por ejemplo, Mexicano, Mexicano Americano, Puertorriqueño, Cubano, Salvadoreño, Dominicano, Colombiano, etc.)

Branching Logic: when "Hispano, Latino o Español" selected, then:

- ❑ Colombiano
- ❑ Cubano
- ❑ Dominicano
- ❑ Ecuatoriano
- ❑ Hondureño
- ❑ Mexicano o mexicano americano
- ❑ Puertorriqueño
- ❑ Salvadoreño
- ❑ Español
- ❑ Ninguna de estas me describe completamente

  Branching Logic: when "Ninguna de estas me describe completamente" selected, then:

  Por favor, especifique.[2]

  - *(texto libre)*

❑ Del Medio Oriente o del Norte de África (por ejemplo, Libanés, Iraní, Egipcio, Sirio, Marroquí, Argelino, etc.)

Branching Logic: when "Del Medio Oriente o del Norte de África" selected, then:

- ❑ Afgano
- ❑ Argelino
- ❑ Egipcio
- ❑ Iraní
- ❑ Iraquí
- ❑ Israelita
- ❑ Libanés
- ❑ Marroquí
- ❑ Sirio
- ❑ Tunecino
- ❑ Ninguna de estas me describe completamente

Branching Logic: when "Ninguna de estas me describe completamente" selected, then:
Por favor, especifique.[2]
- *(texto libre)*

- ❑ Nativo de Hawái o de otras Islas del Pacífico (por ejemplo, Nativo Hawaiano, Samoano, Chamorro, Tongano, Fiyiano, Marshalés, etc.)
  Branching Logic: when "Nativo de Hawái o de otras Islas del Pacífico" selected, then:
  - ❑ Chamorro
  - ❑ Chuukese
  - ❑ Fiyiano
  - ❑ Marshalés
  - ❑ Nativo de Hawái
  - ❑ Palauano
  - ❑ Samoano
  - ❑ Tahitiano
  - ❑ Tongano
  - ❑ Ninguna de estas me describe completamente
    Branching Logic: when "Ninguna de estas me describe completamente" selected, then:
    Por favor, especifique.[2]
    - *(texto libre)*

- ❑ Blanco (por ejemplo, Alemán, Europeo, Irlandés, Inglés, Italiano, Polaco, Francés, etc.)
  Branching Logic: when "Blanco" selected, then:
  - ❑ Holandés
  - ❑ Inglés
  - ❑ Europeo
  - ❑ Francés
  - ❑ Alemán
  - ❑ Irlandés
  - ❑ Italiano
  - ❑ Noruego
  - ❑ Polaco
  - ❑ Escocés
  - ❑ Español
  - ❑ Ninguna de estas me describe completamente
    Branching Logic: when "Ninguna de estas me describe completamente" selected, then:
    Por favor, especifique.[2]
    - *(texto libre)*

- ❑ Ninguna de estas me describe completamente
  Branching Logic: when "Ninguna de estas me describe completamente" selected, then:

Por favor, especifique.[2]
- *(texto libre)*

- ❏ Prefiero no responder

¿Con cuál género se identifica mejor? (Marque todas las que correspondan) [3]

- ❏ Hombre
- ❏ Mujer
- ❏ No binario
- ❏ Transgénero
- ❏ Ninguna de estas opciones me describe y quiero ver opciones adicionales
- ❏ Prefiero no responder

*Branching Logic: when "no binario", "transgénero", or "*ninguna de estas opciones me describe y quiero ver opciones adicionales" *selected, then:*

¿Alguna de estas opciones se acerca más a una descripción de cómo se considera a sí mismo? [3]

- ❏ Hombre transexual/hombre transgénero/mujer a hombre (FTM)
- ❏ Mujer transexual/mujer transgénero/hombre a mujer (MTF)
- ❏ Género no binario
- ❏ Género fluido
- ❏ Variante de género
- ❏ Dos espíritus
- ❏ Con dudas o inseguridad acerca de su identidad de género
- ❏ Ninguna de estas me describe, y quiero especificar

  Branching Logic: when "Ninguna de estas me describe, y quiero especificar" selected, then:

  Por favor, especifique.[3]
  - *(texto libre opcional)*

¿Qué sexo biológico se le asignó al nacer?[3]

- Femenino
- Masculino
- Intersexual
- Ninguno de estos me describe

  Branching Logic: when "Ninguno de estos me describe" selected, then:

  Por favor, especifique.[3]
  - *(respuesta en texto libre)*
- Prefiero no responder

¿Cuál de las siguientes opciones piensa usted que le representa mejor? [4]

- ❏ Gay
- ❏ Lesbiana

- ❏ Heterosexual (no es gay ni lesbiana)
- ❏ Bisexual
- ❏ Ninguna de estas me describe

  Branching Logic: when "Ninguna de estas me describe" selected, then:

  ¿Alguna de estas opciones se acerca más a una descripción de cómo se considera a sí mismo? [5]

  - Queer
  - Polisexual, omnisexual, sapiosexual o pansexual
  - Asexual
  - Dos espíritus
  - No ha descubierto o está en proceso de descubrir su sexualidad
  - Mayormente heterosexual, pero a veces atraído a personas de su mismo sexo
  - No considera que tenga una sexualidad
  - No usa etiquetas para identificarse
  - No sabe la respuesta
  - No, quiero decir otra cosa

    Branching Logic: when "No, quiero decir otra cosa" selected, then:

    Por favor, especifique.[5]

    - *(texto libre opcional)*
- ❏ Prefiero no responder

¿Cuál es el grado o nivel de educación más alto que ha completado? [5]

- Nunca fue a la escuela o solo fue al kínder
- 1° a 4° grado (Primaria)
- 5° a 8° grado (Secundaria)
- 9° a 11° grado (Preparatoria o bachillerato incompleta)
- 12° grado o GED (Preparatoria o bachillerato completo o GED)
- 1 a 3 años de universidad (algunos estudios universitarios o de escuela técnica)
- 4 años o más de universidad (graduado de la universidad)
- Estudios avanzados (Maestría, Doctorado, etc.)
- Prefiero no contestar

¿Alguna vez ha estado en servicio activo en las Fuerzas Armadas de los Estados Unidos, ya sea en el servicio militar regular, en la Guardia Nacional o en una unidad de reserva militar?

NOTA: El servicio activo no incluye el entrenamiento en la Reserva ni en la Guardia Nacional, pero SÍ incluye las actividades de movilización, por ejemplo, para la Guerra del Golfo Pérsico. [5]

- Sí
- No
- Prefiero no contestar

¿Cuál es su estado civil actual? [5]

- Casado/a
- Divorciado/a
- Viudo/a
- Separado/a
- Nunca estuvo casado/a
- Vive en pareja sin estar casado/a / unión libre
- Prefiero no contestar

Las siguientes dos preguntas son sobre las personas que viven con usted.

Sin contarse usted, ¿cuántas personas viven con usted en su casa? [5]

- *Texto libre (valor entero)*

  Branching Logic: when anything other than "0" is entered, then:

  Entre las personas que viven con usted, ¿cuántos son menores de 18 años? [1]

  - *Texto libre (valor entero)*

Las siguientes preguntas serán sobre su seguro médico. Incluya el seguro médico que usted tiene por su trabajo o que usted compra directamente y también los programas del gobierno como Medicare y Medicaid que proveen atención médica o que le ayudan a pagar las facturas médicas.

¿Tiene usted seguro médico o algún tipo de plan para el cuidado de su salud? [1]

- Sí

  Branching Logic: when "Sí" selected, then:

  Actualmente ¿está cubierto por algún seguro médico o planes de cobertura médica?[2]

  - ❑ Seguro a través de su trabajo previo o actual o a través de una asociación de trabajadores (de parte de usted u otro familiar)
  - ❑ Seguro comprado directamente a una compañía de seguros (de parte de usted u otro familiar)
  - ❑ Medicare para personas de 65 años o más o con ciertas discapacidades
  - ❑ Medicaid, ayuda médica o algún tipo de plan de ayuda del gobierno para personas con bajos ingresos o discapacidades
  - ❑ TRICARE o alguna otra atención médica para militares
  - ❑ Departamento de Asuntos para Veteranos (VA, por sus siglas en inglés) (incluye los que alguna vez ha usado o se ha inscrito para la atención médica de VA)
  - ❑ Servicio Médico para Indio Americano
  - ❑ Otro tipo de seguro médico o plan de cobertura médica

Branching Logic: when "Otro tipo de seguro médico o plan de cobertura médica" selected, then:

Por favor, especifique:[2]

texto libre

❑ No tengo seguro médico, yo pago por mi cuenta

- No
- No sé
- Prefiero no responder

Las siguientes preguntas son para saber si usted tiene alguna discapacidad. En algunas de ellas se le preguntará sobre más de una discapacidad a la vez. Por favor conteste "Sí" si usted tiene al menos una de ellas. *(Optional Informational Pop-up: "El Congreso de los Estados Unidos aprobó una ley para proteger a las personas con discapacidad. Esa ley se llama Ley sobre Estadounidenses con Discapacidades (ADA, por sus siglas en inglés. Tener una discapacidad significa que usted podría tener un problema físico o mental. Este problema podría dificultarle hacer ciertas cosas. Podría tener problemas al caminar, respirar, aprender, leer, hablar, ver, escuchar o pensar.")*

¿Es usted sordo/a o tiene serias dificultades al escuchar?[10]

- Sí
- No
- Prefiero no responder

¿Es usted ciego/a o tiene serias dificultades para ver, incluso mientras usa lentes/anteojos?[10]

- Sí
- No
- Prefiero no responder

¿Tiene usted alguna discapacidad física, mental o emocional que le hace difícil concentrarse, recordar o tomar decisiones?[10]

- Sí
- No
- Prefiero no responder

¿Tiene usted serias dificultades para caminar o subir escaleras?[10]

- Sí
- No
- Prefiero no responder

¿Tiene usted dificultades para vestirse o bañarse?[10]

- Sí
- No
- Prefiero no responder

¿Tiene usted alguna discapacidad física, mental o emocional que le dificulta completar sus tareas cotidianas, tales como visitar la oficina de su médico o ir de compras?[10]

- Sí
- No
- Prefiero no responder

Las siguientes preguntas son sobre su trabajo, ingreso, y de donde vive.

¿Cuál es su situación de trabajo actual?[5]

- ❏ Empleado/a asalariado/a (tiempo parcial o tiempo completo)
- ❏ Trabajador/a independiente
- ❏ Desempleado/a por 1 año o más
- ❏ Desempleado/a por menos de 1 año
- ❏ Soy una mujer u hombre que me ocupo de tareas de la casa/ ama de casa
- ❏ Estudiante
- ❏ Jubilado/a
- ❏ No puede trabajar (por discapacidad)
- ❏ Prefiero no contestar

Branching Logic: when "Empleado/a asalariado/a (tiempo parcial o tiempo completo)" o "Trabajador/a independiente" selected, then:

Al compartir información donde usted trabaja, podría ayudarnos a entender cómo el medio ambiente afecta la salud. Compartir la dirección de su trabajo es su decisión. Usted puede decir no, y aun así seguir participando en el programa.

¿Si recibe ingresos por tener empleo autónomo o de una empresa, cual es la dirección de su empleo?[6]

- ○ Dirección

Branching Logic: when "Enter Address" selected, then:

Dirección postal[6]

____

Dirección - línea adicional (opcional)[6]

____

Ciudad[6]

____

Estado[6]

____

Código postal[6]

____

País[6]

____

○ Prefiero no contestar

Una de las cosas que intentamos entender es cómo los ingresos de las personas afectan el uso de los servicios de salud. El ingreso familiar incluye sus ingresos más los de todos los integrantes de su familia en su hogar durante el último año de calendario. Incluya todos los salarios y otras fuentes de ingreso.

¿Cuál es su ingreso familiar anual incluyendo otras fuentes?[5]

- Menos que $10.000
- $10,000- $24,999
- $25,000- $34,999
- $35,000- $49,999
- 50,000- $74,999
- $75,000-$99,999
- $100,000- $149,999
- $150,000- $199,999
- $200,000 o más
- Prefiero no responder

¿Vive usted en casa propia o alquilada (rentada)?[7]

- Propia
- Alquilada (rentada)
- Otra situación de vivienda

  Branching Logic: when "Otra situación de vivienda" selected, then:

  ¿En dónde vive actualmente?[7]

  - En un campo universitario
  - Con un amigo/compañero de habitación
  - Con familia
  - Motel/hotel
  - Hospital, centro de rehabilitación, centro de tratamiento por abuso de drogas u otra institución temporal
  - En una casa comunitaria, un asilo u otro centro residencial
  - Hogar de transición

  - ○ Albergue de emergencia o para personas sin hogar
  - ○ En cualquier lugar afuera (por ejemplo, calle, vehículo, edificio abandonado)
  - ○ Otro

    Branching Logic: when "Otro" selected, then:

    Por favor, especifique.[7]

    (texto libre)

- Prefiero no contestar

¿Cuántos años ha vivido en su dirección actual?[8]

- Menos de 1 año
- 1-2 años
- 3-5 años
- 6-10 años
- 11-20 años
- Más de 20 años

La siguiente pregunta es sobre el estrés que usted puede sentir sobre el dinero.

En los últimos 6 meses, ¿ha estado usted preocupado por NO tener un lugar estable donde vivir del que sea dueño, que rente (alquile) o se aloje como parte de un hogar?[9]

- Sí
- No

Si usted tiene un número de seguro social, y decide compartirlo con nosotros podría contribuir para agregar información adicional a la base de datos de All of Us. Esta información podría venir de lugares como su proveedor de salud o farmacia. Compartir su número de seguro social es su decisión. Usted puede decir no y aun así seguir participando en el programa.

¿Cuál es su número de seguro social?[6]

- Número de seguro social

  Branching Logic: when "Número de seguro social" selected, then:

  Número de seguro social[6]

  ____

- Prefiero no contestar

En esta última sección se solicita información de contacto de su familia o amigos para que

podamos mantener la comunicación con usted en el futuro. Esta información no es necesaria para poder participar en el programa. Toda la información que usted nos da se guardará de forma segura.

El Programa Científico All of Us se pondrá en contacto con usted periódicamente para reunir información adicional relacionada con su salud. Por favor denos los nombres, direcciones y números de teléfono de 2 familiares o amigos que no viven con usted en caso de que no lo podamos contactar. (Por favor, denos los nombres de personas que actualmente no viven en su casa).[1]

- **Nombre de la primera persona**[1]

  ____
- **Inicial del segundo nombre de la primera persona**[1]

  ____
- **Apellido de la primera persona**[1]

  ____
- **Dirección número 1 de la primera persona**[1]

  ____
- **Dirección número 2 de la primera persona**[1]

  ____
- **Estado de primera persona**[1]

  ____
- **Código postal de primera persona**[1]

  ____
- **Correo electrónico de primera persona**[1]

  ____
- **Número de teléfono de primera persona**[1] *(no permitir prefiero no contestar o no sé)*

  ____
- **Relación con el entrevistado**[1]
  - ○ Hijo
  - ○ Amigo
  - ○ Padre/custodio legal
  - ○ Pariente
  - ○ Esposo/cónyuge
- **Nombre de la segunda persona**[1]

  ____
- **Inicial del segundo nombre de la segunda persona**[1]

  ____
- **Apellido de la segunda persona**[1]

  ____
- **Dirección número 1 de la segunda persona**[1]

  ____

- **Dirección número 2 de la segunda persona**[1]

  ____

- **Estado de la segunda persona**[1]

  ____

- **Código postal de la segunda persona**[1]

  ____

- **Correo electrónico de la segunda persona**[1]

  ____

- **Número de teléfono de la segunda persona**[1] (no permitir prefiero no contestar o no sé)

  ____

- **Relación con el entrevistado**[1]
  - Hijo
  - Amigo
  - Padre/custodio legal
  - Pariente
  - Esposo/cónyuge

All of Us Research Program
Participant Provided Information (PPI)
Version: October 21, 2019

Sources

1. National Health and Nutrition Examination Survey (NHANES)
2. U.S. 2020 Census (2015 Draft)
3. GenIUSS group (Gender Identity in U.S. Surveillance)
4. National Health Interview Survey (NHIS)
5. Behavioral Risk Factor Surveillance System (BRFSS)
6. Developed for use in *All of Us*
7. National Health Care for the Homeless Council (NHCHC)
8. UK Biobank
9. VA Homelessness Screening Clinical Reminder (HSCR)
10. American Community Survey (ACS) (added to Basics October 2019)
