# Supplementary material for: Racial, ethnic, and gender differences in obesity and body fat distribution: An All of Us Research Program demonstration project
Source: PLoS One. 2021 Aug 6;16(8):e0255583. doi: 10.1371/journal.pone.0255583 (PMC8345840; doi:10.1371/journal.pone.0255583)
Supplement: S4 File — (PDF) [file pone.0255583.s004.pdf]

All of Us Research Program
Participant Provided Information (PPI)
Version: May 30, 2017

Lifestyle

This survey asks questions about your use of tobacco, alcohol, and drugs. This is to better understand how these things may affect your overall health. Your privacy is very important to us. Your name will be separated from your answers before they are shared with researchers.

It takes about 5-10 minutes to answer these questions. Please answer each question as honestly as possible. It is important that you answer as many questions as you can. We are looking for your own answers, and not what you think your doctors, family, or friends want you to say.

Don't feel like you have to spend a long time over each question. The first answer that comes to you is usually the best one. If you aren't sure how to answer a question, choose the best answer from the options given.

Have you smoked at least 100 cigarettes in your entire life? (There are 20 cigarettes in a pack.)?[1–3]

- Yes
- No
- Don't know
- Prefer not to answer

  Branching Logic if "Yes" selected, display the following:

  Do you now smoke cigarettes every day, some days, or not at all?[1–3]
    - Every day
    - Some days
    - Not at all
    - Don't know
    - Prefer not to answer

  How old were you when you first started regular cigarette smoking?[1–3]
    - Enter Age: ____________ [Range 1-99]
    - Don't know
    - Prefer not to answer

  In the past, have you ever made a serious attempt to quit smoking? That is, have you stopped smoking for at least one day or longer because you were trying to quit?[1–3]
    - Yes
    - No
    - Don't know
    - Prefer not to answer

      Branching Logic if "yes" selected, display the following:

      If you have completely stopped smoking cigarettes, how old were you when you stopped?[2,4]

- _______________ Age Stopped Smoking
- Don't know
- Prefer not to answer

How many years have you or did you smoke cigarettes?[2,4]

- _______________ Years (0-99)
- Don't Know
- Prefer not to answer

On average, how many cigarettes do you smoke per day now? (There are 20 cigarettes in a pack.)[1–3]

- Enter number of cigarettes per day _________ [Range: 1-99]
- Don't know
- Prefer not to answer

On average, over the entire time that you smoked, how many cigarettes did you smoke each day? (There are 20 cigarettes in a pack.)[1–3]

- Enter number of cigarettes per day _________ [Range: 1-99]
- Don't know
- Prefer not to answer

Have you ever used an electronic nicotine product, even one or two times? (Electronic nicotine products include e- cigarettes, vape pens, hookah pens, personal vaporizers and mods, e-cigars, e-pipes, and e-hookahs.)[5]

- Yes
- No
- Don't Know
- Prefer Not To Answer

Branching Logic if "yes" selected, display the following:

Do you now use electronic nicotine products…[5]

- Every day
- Somedays
- Not at all
- Don't know
- Prefer not to answer

Have you ever smoked a traditional cigar, cigarillo, or filtered cigar, even one of two puffs?[5]

- Yes
- No
- Don't Know
- Prefer Not To Answer

Branching Logic if "yes" selected, display the following:

Do you now smoke a traditional cigar, cigarillo, or filtered cigar…[5]

- Every day
- Somedays
- Not at all

- Don't know
- Prefer not to answer

Have you ever smoked tobacco in a hookah, even one or two puffs?[5]

- Yes
- No
- Don't Know
- Prefer Not To Answer

  Branching Logic if "yes" selected, display the following:

  Do you smoke hookah…

  - Every day
  - Somedays
  - Not at all
  - Don't know
  - Prefer not to answer

Have you ever used smokeless tobacco products, even one or two times? (Smokeless tobacco products include snus pouches, Skoal Bandits, loose snus, moist snuff, dip, spit, and chewing tobacco.)[5]

- Yes
- No
- Don't Know
- Prefer Not To Answer

  Branching Logic if "yes" selected, display the following:

  Do you now use smokeless tobacco products…[5]

  - Every day
  - Somedays
  - Not at all
  - Don't know
  - Prefer not to answer

Thanks for your answers. The next questions will ask about drinking alcohol. This includes coolers, beer, wine, champagne, liquor such as whiskey, rum, gin, vodka, scotch, or liqueurs, and also any other type of alcohol. This will help researchers better understand how alcohol affects health. As always, your answers are private.

In your entire life, have you had at least 1 drink of any kind of alcohol, not counting small tastes or sips? (By a "drink," we mean a can or bottle of beer, a glass of wine or a wine cooler, a shot of liquor, or a mixed drink with liquor in it.)[6]

- Yes
- No
- Prefer Not To Answer

  Branching Logic if 'yes' selected:

How often did you have a drink containing alcohol in the past year?[7]

- Never
- Monthly or less
- Two to four times a month
- Two to three times a week
- Four or more times a week
- Prefer not to answer

Branching Logic if anything other than 'Never' or 'prefer not to answer' selected:

On a typical day when you drink, how many drinks do you have?[7]

- 1 or 2
- 3 or 4
- 5 or 6
- 7 to 9
- 10 or more
- Prefer not to answer

How often did you have six or more drinks on one occasion in the past year?[7]

- Less than monthly
- Monthly
- Weekly
- Daily or almost daily
- Never in the last year
- Prefer not to answer

Thanks for your answers. Now we'd like to ask you about your experiences with medicines and other kinds of drugs. Some of the substances we'll talk about are prescribed by a doctor (like pain medications). We only want to know if you have taken them for reasons or in doses other than prescribed. We understand that these are sensitive questions. You may choose not to answer them. However, by providing answers, you are helping researchers better understand how these substances affect health.

In your LIFETIME, which of the following substances have you ever used?[8]

- Marijuana (cannabis, pot, grass, hash, weed, etc.)
- Cocaine (coke, crack, etc.)
- Prescription stimulants for non-medical reasons (Ritalin, Concerta, Dexedrine, Adderall, diet pills, etc.)
- Other stimulants (methamphetamine, speed, crystal meth, ice, k2/spice, bath salts, etc.)
- Inhalants (nitrous oxide, glue, gas, paint thinner, etc.)
- Sedatives or sleeping pills for non-medical reasons (Valium, Serepax, Ativan, Xanax, Librium, Rohypnol, GHB, etc.)
- Hallucinogens (LSD, acid, mushrooms, PCP, Special K, ecstasy, etc.)
- Street opioids (heroin, opium, etc.)

- Prescription opioids for non-medical reasons (fentanyl, oxycodone [OxyContin, Percocet], hydrocodone [Vicodin], methadone, buprenorphine, etc.)
- Other (Specify)
  Branching Logic for substance with "Yes" responses, display the following:
  In the PAST THREE MONTHS, how often have you used marijuana (cannabis, pot, grass, hash, etc.)?
    - o Never
    - o Once or twice
    - o Monthly
    - o Weekly
    - o Daily or almost daily
    - o Prefer not to answer

  In the PAST THREE MONTHS, how often have you used cocaine (coke, crack, etc.)?
    - o Never
    - o Once or twice
    - o Monthly
    - o Weekly
    - o Daily or almost daily
    - o Prefer not to answer

  In the PAST THREE MONTHS, how often have you used prescription stimulants for non-medical reasons (Vyvanse, Ritalin, Concerta, Dexedrine, Adderall, diet pills, etc.)
    - o Never
    - o Once or twice
    - o Monthly
    - o Weekly
    - o Daily or almost daily
    - o Prefer not to answer

  In the PAST THREE MONTHS, how often have you used other stimulants (methamphetamine, speed, crystal meth, ice, k2/spice, bath salts, etc.)
    - o Never
    - o Once or twice
    - o Monthly
    - o Weekly
    - o Daily or almost daily
    - o Prefer not to answer

  In the PAST THREE MONTHS, how often have you used inhalants (nitrous oxide, glue, gas, paint thinner, etc.)?
    - o Never
    - o Once or twice
    - o Monthly

- o Weekly
- o Daily or almost daily
- o Prefer not to answer

In the PAST THREE MONTHS, how often have you used sedatives or sleeping pills for non-medical reasons (Valium, Serepax, Ativan, Xanax, Librium, Rohypnol, GHB, etc.)?

- o Never
- o Once or twice
- o Monthly
- o Weekly
- o Daily or almost daily
- o Prefer not to answer

In the PAST THREE MONTHS, how often have you used hallucinogens (LSD, acid, mushrooms, PCP, Special K, ecstasy, etc.)?

- o Never
- o Once or twice
- o Monthly
- o Weekly
- o Daily or almost daily
- o Prefer not to answer

In the PAST THREE MONTHS, how often have you used street opioids (heroin, opium, etc.)?

- o Never
- o Once or twice
- o Monthly
- o Weekly
- o Daily or almost daily
- o Prefer not to answer

In the PAST THREE MONTHS, how often have you used prescription opioids for non-medical reasons (obana, fentanyl, oxycodone [OxyContin, Percocet], hydrocodone [Vicodin], methadone, buprenorphine [Supoxone], etc.)

- o Never
- o Once or twice
- o Monthly
- o Weekly
- o Daily or almost daily
- o Prefer not to answer

In the PAST THREE MONTHS, how often have you used other drugs?

- o Never
- o Once or twice
- o Monthly

- o Weekly
- o Daily or almost daily
- o Prefer not to answer

Thank you for answering these questions. We know they are very personal.
We want to remind you that your answers will only be shared with approved researchers.
Your privacy is very important to us.

<u>Sources</u>

1. Tobacco Use Supplement to the Current Population Survey (TUS-CPS)
2. American Thoracic Society Division of Lung Disease questionnaire (ATS – DLD-78)
3. Million Veterans Program
4. Prostate, Lung, Colorectal and Ovarian (PLCO) Cancer Screening Trial
5. Population Assessment of Tobacco and Health (PATH)
6. National Epidemiologic Survey on Alcohol and Related Conditions (NESARC)
7. Alcohol Use Disorders Identification Test (AUDIT-C)
8. NM ASSIST (NIDA-Modified Alcohol, Smoking, and Substance Involvement Screening Test)
