# Supplementary material for: Racial, ethnic, and gender differences in obesity and body fat distribution: An All of Us Research Program demonstration project
Source: PLoS One. 2021 Aug 6;16(8):e0255583. doi: 10.1371/journal.pone.0255583 (PMC8345840; doi:10.1371/journal.pone.0255583)
Supplement: S5 File — (PDF) [file pone.0255583.s005.pdf]

Estilo de Vida

Esta encuesta le hace preguntas sobre su consumo de tabaco, alcohol y drogas. Esta información nos sirve para comprender mejor cómo estos aspectos pueden afectar su salud. Para asegurar su privacidad, su nombre se separará de sus respuestas antes de compartirlas con los investigadores.

Responder a las siguientes preguntas le llevará como 5-10 minutos. Por favor responda cada pregunta lo más honesto posible. Para cualquiera de estas preguntas no hay respuestas correctas o incorrectas. Es importante que responda todas las preguntas que pueda. Estamos buscando sus propias respuestas, y no lo que piensan sus doctores, familiares, o amigos.

No sienta que tiene que tomar mucho tiempo para cada pregunta. La primera respuesta que se le ocurre es usualmente la mejor. Si no está seguro(a) de cómo responder la pregunta, escoja la mejor respuesta de las opciones presentadas.

¿Ha fumado usted al menos 100 cigarrillos en toda su vida? (Cada paquete tiene 20 cigarrillos)

- Sí
- No
- No sé
- Prefiero no contester

  Branching Logic if "Sí", display the following:

  Actualmente, ¿Con qué frecuencia fuma usted cigarrillos?
  - Todos los días
  - Algunos días
  - Nunca
  - No sé
  - Prefiero no contestar

  ¿Qué edad tenía usted cuando empezó a fumar cigarrillos regularmente?
  - Anote la edad: ____ [RANGO: 1 - 99]
  - No sé
  - Prefiero no contestar

  En el pasado, ¿alguna vez ha intentado seriamente dejar de fumar? Es decir, ¿ha dejado de fumar durante un día o más porque estaba tratando de dejar de fumar?
  - Sí
  - No
  - No sé
  - Prefiero no contestar

    Branching Logic if "Sí", display the following:

    Si ha dejado por completo de fumar cigarrillos, ¿qué edad tenía cuando lo dejó?
    - ___ EDAD A LA QUE DEJÓ DE FUMAR

- No sé
- Prefiero no responder

¿Durante cuántos años fumó cigarrillos?

- ____ AÑOS como fumador/a [escala: 1-99]
- No sé
- Prefiero no responder

En promedio, ¿cuántos cigarrillos fuma al día actualmente (una caja contiene 20 cigarrillos)?

- cigarrillos por día _____ [escala: 1-99]
- No sé
- Prefiero no responder

Durante todo el tiempo que fumó, ¿cuántos cigarrillos fumaba en promedio por día (una caja contiene 20 cigarrillos)?

- cigarrillos por día _____ [escala: 1-99]
- No sé
- Prefiero no responder

¿Alguna vez usó un producto electrónico con nicotina, aunque sea una o dos veces? (Cigarrillos electrónicos, cigarrillos a vapor, hookah, vaporizadores y módulos personales, puros electrónicos, pipas electrónicas y hookah electrónica)

- Sí
- No
- No sé
- Prefiero no responder

Branching Logic if "Sí", display the following:

Actualmente, ¿usa productos electrónicos con nicotina?

- Todos los días
- Algunos días
- Nunca
- No sé
- Prefiero no responder

¿Alguna vez fumó un puro o cigarrillo tradicional o puro con filtro, aunque sea una o dos fumadas para probar?

- Sí
- No
- No sé
- Prefiero no responder

Branching Logic if "Sí", display the following:

Actualmente, ¿fuma puros o cigarrillos tradicionales con o sin filtro?

- Todos los días
- Algunos días

- Nunca
- No sé
- Prefiero no responder

¿Alguna vez fumó tabaco en hookah, aunque sea una o dos caladas (probadas, chupadas, fumadas)?

- Sí
- No
- No sé
- Prefiero no responder

  Branching Logic if "Sí", display the following:

  Actualmente, ¿fuma hookah?

  - Todos los días
  - Algunos días
  - Nunca
  - No sé
  - Prefiero no responder

¿Alguna vez usó productos de tabaco sin humo, aunque sea una o dos veces? (snus, Skoal Bandits, snus suelto, rapé húmedo, tabaco para masticar y escupir)

- Sí
- No
- No sé
- Prefiero no responder

  Branching Logic if "Sí", display the following:

  Actualmente, ¿consume productos de tabaco sin humo?

  - Todos los días
  - Algunos días
  - Nunca
  - No sé
  - Prefiero no responder

Gracias por sus respuestas. Las siguientes preguntas serán sobre su consumo de alcohol. Esto incluye refresco con alcohol, cerveza, vino, champán, licor como whisky, ron, ginebra (gin), vodka, escocés (scotch), o licores, y también todo otro tipo de alcohol.

Esto ayudará para que los investigadores comprendan mejor cómo el alcohol afecta la salud. Como siempre, sus respuestas son privadas.

¿Alguna vez en su vida consumió al menos una bebida alcohólica de cualquier tipo, sin contar pequeñas probadas? (Con "bebida" nos referimos a una lata o botella de cerveza, una copa de vino o refrescos de vino, un trago (shot) de licor o una bebida mezclada con licor.)

- Sí
- No
- Prefiero no responder

  Branching Logic if "Sí" display the following:

  ¿Con qué frecuencia consumió bebidas alcohólicas en el último año?
  - Nunca
  - Una vez al mes o menos
  - De dos a cuatro veces al mes
  - De dos a tres veces a la semana
  - Cuatro veces o más a la semana

    Branching Logic if anything greater than "Nunca" display the following:

    Durante el año pasado, ¿cuántas bebidas tomaba en un día común?
    - 1 o 2
    - 3 o 4
    - 5 o 6
    - 7 a 9
    - 10 o más
    - Prefiero no responder

    ¿Cuántas veces tomó seis o más bebidas en una ocasión durante el último año?
    - Menos de una vez al mes
    - Una vez al mes
    - Una vez a la semana
    - A diario o casi todos los días
    - Nunca en el último año
    - Prefiero no responder

Gracias por sus respuestas. Ahora nos gustaría preguntarle sobre sus experiencias con medicinas y otros tipos de drogas. Algunas de las sustancias de las que vamos a hablar son recetadas por un doctor (como medicamentos para el dolor). Nosotros sólo queremos saber si las ha tomado por razones diferentes a las indicadas o en dosis diferentes de las que han sido recetadas. Comprendemos que estas son preguntas delicadas. Usted puede elegir no responderlas. Sin embargo, al proporcionar sus respuestas ayudará a los investigadores a comprender mejor cómo estas sustancias afectan a la salud.

En TODA SU VIDA, ¿cuál de estas sustancias ha usado alguna vez?
- Marihuana (cannabis, mota, hachís, hierba, etc.)
- Cocaína (coca, crack, etc.)
- Estimulantes recetados para fines no médicos (Ritalin, Concerta, Dexedrine, Adderall, píldoras para adelgazar, etc.)
- Otros estimulantes (metanfetamina, tachas, anfetaminas, cristal, spice, sales de baño, etc.)

- Inhalantes (óxido nitroso, pegamento, gas, thinner o diluyente de pintura, etc.)
- Sedantes o píldoras para dormir para fines no médicos (Valium, Serepax, Ativan, Xanax, Librium, Rohypnol, GHB, etc.)
- Alucinógenos (LSD, ácido, hongos, PCP, K especial, éxtasis, etc.)
- Opioides obtenidos en la calle (heroína, opio, etc.)
- Opioides recetados para fines no médicos (fentanilo, oxicodona [OxyContin, Percocet], hidrocodona [Vicodin], metadona, buprenorfina, etc.)
- Ninguna de estas drogas
- Prefiero no responder
- Otro (especifique)

Branching Logic for substance with "Sí" responses, display the following:

En los últimos tres meses, ¿con qué frecuencia usó las sustancias que mencionó de marihuana (cannabis, mota, hachís, hierba, etc.)?

- Nunca
- Una o dos veces
- Una vez al mes
- Una vez a la semana
- A diario o casi todos los días
- Prefiere no responder

En los últimos tres meses, ¿con qué frecuencia usó las sustancias que mencionó de cocaína (coca, crack, etc.)?

- Nunca
- Una o dos veces
- Una vez al mes
- Una vez a la semana
- A diario o casi todos los días
- Prefiere no responder

En los últimos tres meses, ¿con qué frecuencia usó las sustancias que mencionó de estimulantes recetados por motivos no médicos (Vyvanse Ritalin, Concerta, Dexedrina, Adderall, píldoras para bajar de peso, etc.)?

- Nunca
- Una o dos veces
- Una vez al mes
- Una vez a la semana
- A diario o casi todos los días
- Prefiere no responder

En los últimos tres meses, ¿con qué frecuencia usó las sustancias que mencionó de (metanfetamina [meta speed, cristalizada, solidificada, etc.], k2/spice, sales de baño)?

- Nunca
- Una o dos veces
- Una vez al mes

- Una vez a la semana
- A diario o casi todos los días
- Prefiere no responder

En los últimos tres meses, ¿con qué frecuencia usó las sustancias que mencionó de inhalantes (óxido nitroso, pegamento, gasolina, diluyente de pintura, etc.)?

- Nunca
- Una o dos veces
- Una vez al mes
- Una vez a la semana
- A diario o casi todos los días
- Prefiere no responder

En los últimos tres meses, ¿con qué frecuencia usó las sustancias que mencionó de sedantes o píldoras para dormir por motivos no médicos (Valium, Serepax, Ativan, Xanax, Librium, Rohypnol, GHB, etc.)?

- Nunca
- Una o dos veces
- Una vez al mes
- Una vez a la semana
- A diario o casi todos los días
- Prefiere no responder

En los últimos tres meses, ¿con qué frecuencia usó las sustancias que mencionó de alucinógenos (LSD, ácidos, hongos, PCP, ketamina, éxtasis, etc.)?

- Nunca
- Una o dos veces
- Una vez al mes
- Una vez a la semana
- A diario o casi todos los días
- Prefiere no responder

En los últimos tres meses, ¿con qué frecuencia usó las sustancias que mencionó de opioides callejeros (heroína, opio, etc.)?

- Nunca
- Una o dos veces
- Una vez al mes
- Una vez a la semana
- A diario o casi todos los días
- Prefiere no responder

¿En los ÚLTIMOS TRES MESES, con qué frecuencia ha usado opioides recetados por un doctor por razones no médicas (obana, fentanyl, oxicodona [OxyContin, Percocet], hidrocodona [Vicodin], metadona, buprenorfina [Supoxone, etc.)?

- Nunca
- Una o dos veces
- Una vez al mes
- Una vez a la semana

- A diario o casi todos los días
- Prefiere no responder

¿En los ÚLTIMOS TRES MESES, con qué frecuencia ha usado otras drogas?

- Nunca
- Una o dos veces
- Una vez al mes
- Una vez a la semana
- A diario o casi todos los días
- Prefiere no responder

Gracias por responder estas preguntas. Sabemos que son muy personales. Queremos recordarle que sus respuestas sólo se compartirán con los investigadores autorizados. Su privacidad es muy importante para nosotros.
